# Genome-Wide Association Mapping for Salt Tolerance of Rice Seedlings Grown in Hydroponic and Soil Systems Using the Bengal and Assam Aus Panel

**DOI:** 10.3389/fpls.2020.576479

**Published:** 2020-10-23

**Authors:** Caijin Chen, Gareth J. Norton, Adam H. Price

**Affiliations:** School of Biological Science, University of Aberdeen, Aberdeen, United Kingdom

**Keywords:** salinity tolerance, quantitative trait locus, genome-wide association mapping, seedling stage, *Oryza sativa*, *aus*

## Abstract

Salinity is a major abiotic stress which inhibits rice production in coastal, arid and semi-aid areas in many countries, such as India and Bangladesh. Identification of salt tolerant cultivars, quantitative trait loci (QTLs) and genes is essential for breeding salt tolerant rice. The *aus* subpopulation of rice is considered to have originated predominantly from Bangladesh and India and have rich genetic diversity with wide variation in abiotic stress resistance. The objective of this study was to identify QTLs, and subsequently candidate genes using cultivars from the *aus* subpopulation and compare the results of two different seedling stage screening methods. Salt tolerance at the rice seedling stage was evaluated on 204 rice accessions from the Bengal and Assam Aus Panel (BAAP) grown in both hydroponics and soil under control and salt stress conditions. Ten salt related traits of stress symptoms, plant growth and the content of sodium and potassium were measured. Three cultivars, BRRI dhan 47, Goria, and T 1 showed more salt tolerance than the tolerant check Pokkali in both systems. Genome-wide association mapping was conducted on salt indices traits with 2 million SNPs using an efficient mixed model (EMMA) controlling population structure and kinship, and a significance threshold of *P* < 0.0001 was used to determine significant SNPs. A total of 97 and 74 QTLs associated with traits in hydroponic and soil systems were identified, respectively, including 11 QTLs identified in both systems. A total of 65 candidate genes were found including a well-known major gene *OsHKT1;5*. The most significant QTL was detected at around 40 Mb on chromosome 1 coinciding with two post-translational modifications SUMOylation genes (*OsSUMO1* and *OsSUMO2*), this QTL was investigated. The salt tolerance rice cultivars and QTLs/genes identified here will provide useful information for future studies on genetics and breeding salt tolerant rice.

## Introduction

Rice (*Oryza sativa* L.) is one of the most heavily consumed crops and makes a large contribution to global food security (Lee et al., 2007). According to FAO (2016), world cereal production in 2016 was approximately 3,417 mt (million tons) out of which 27.9% was rice (952 mt). Abiotic stresses like salinity are a major threat for food security. Soil salinity and sodicity is increasing globally due to climate change, increased use of chemical fertilizers, and application of unsuitable irrigation water (Tian et al., 2011). It was estimated that approximately 950 million hectares of arable land globally, including 250 million hectares of irrigated land, is affected by salinity (Solis et al., 2020). Rice is salt sensitive and its threshold for salt stress is electrical conductivity (EC) 3 dS m^–1^ in soils (Negrão et al., 2011). Salt stress affects rice productivity worldwide especially in coastal and inland areas (Batayeva et al., 2018). For example, Dasgupta et al. (2014) reported that before 2050, the productivity of rice will decline of 15.6% in nine subdistricts in coastal Bangladesh where soil salinity of EC is estimated will exceed 4 dS m^–1^.

To develop salinity tolerant cultivars, breeders need simple and efficient mass screening techniques, access to adequate genetic variability, and an understanding of the physiological and genetic control of tolerance (Negrão et al., 2011). Screening under field conditions is difficult due to stress heterogeneity, the presence of other soil-related stresses, and the influence of weather-related factors such as temperature and relative humidity (Gregoria et al., 1997). Hydroponic screening is fast, easy to control, and well adapted to the high volume of materials coming from breeding programs. But in such conditions, transpiration is too low to be representative of field conditions, and the imposed stress is either often not gradual enough or is too severe (Flowers and Colmer, 2008). Soil-based screening as a compromise between hydroponic and field has several advantages, since it allows control of soil, water and “weather” conditions, and like hydroponics, it facilitates evaluation of a large number of genotypes (Sikirou et al., 2016).

On exposing rice plants to salt stress, they suffer a decrease in stomatal conductance, transpiration rate and plant growth (Munns, 2002; Moradi and Ismail, 2007). The uptake of Na^+^ ions by plant roots activates perception and signaling mechanisms, that tend to limit further uptake of Na^+^ ions, reduce Na^+^ transport from root to shoot and finally, restores leaf ion homeostasis (Negrão et al., 2011). The influx of Na^+^ into roots and its movement toward leaves strongly competes with K^+^ uptake, activating ion transport systems that have high affinities for K^+^ and low affinities for Na^+^ (Maathuis et al., 1996). Salinity tolerance is a complex trait controlled by quantitative trait loci (QTLs). Many QTLs associated with the salt related traits including Na^+^ and K^+^ content within roots and shoots have been identified in rice in bi-parental mapping populations (Koyama et al., 2001; Bonilla et al., 2002; Lin et al., 2004; Ming-Zhe et al., 2005; Lee et al., 2007; Sabouri and Sabouri, 2008; Ammar et al., 2009; Pandit et al., 2010; Islam et al., 2011; Ghomi et al., 2013; Mohammadi et al., 2013; Hossain et al., 2015). For example, the SALTOL locus was mapped from recombinant inbred lines which were developed from an *indica* cross between the salt-tolerant cultivar Pokkali and the salt-susceptible cultivar IR29 (Bonilla et al., 2002). Moreover, some salt tolerance-associated genes of rice were cloned by map-based cloning and studied in depth (Ren et al., 2005). For instance, the *SKC1* (*OsHKT1;5*) gene regulating K^+^/Na^+^ homeostasis in the salt-tolerant *indica* rice cultivar Nona Bokra has been cloned using a map-based approach (Ren et al., 2005).

Asian rice cultivars have been broadly classified into five subpopulations: *indica, aus, temperate japonica, tropical japonica* and a*romatic* based on genetic variation (Garris et al., 2005; Zhao et al., 2010; Kim et al., 2016) and geographical distribution (Kim et al., 2016). The *aus* subpopulation of rice originated predominantly from Bangladesh and North East India, it has a large genetic diversity and is grown under a range of conditions from fully irrigated to upland (Glaszmann, 1987; Ali et al., 2013). Whole genome sequencing of cultivated and wild rice cultivars suggested that the cultivated *aus* subpopulation is most closely related to the annual wild rice *O. nivara* and represents a pool of genetic diversity that has been poorly exploited to date (Kim et al., 2016). With the advent of rapid and extensive DNA markers, this genetic diversity can be explored using genome-wide association studies (GWAS) which can assay multiple alleles within a specie, and identify the location of genes affecting traits with high accuracy (Huang et al., 2010). GWAS has been applied to salt stress tolerance in rice in 12 studies mostly using hydroponics (Kumar et al., 2015; Shi et al., 2017; Yu et al., 2017; Campbell et al., 2017; Batayeva et al., 2018; Cui et al., 2018; Frouin et al., 2018; Patishtan et al., 2018; Lekklar et al., 2019; Liu et al., 2019; Rohila et al., 2019; An et al., 2020). None have used both hydroponics and soil (although one has used hydroponics and the field; Liu et al., 2019) and none have included enough *aus* cultivars to assess that subpopulation. In this study, we performed GWAS for identification of potential genes associated with salinity stress tolerance in *aus* rice using the Bengal and Assam Aus Panel (BAAP) by correlating the genotyping information (2 million SNP markers) with the phenotypic traits. The objectives of this study were (1) to screen novel germplasm for salinity-tolerance to identify tolerant cultivars; (2) to identify novel QTLs/genes associated with seedling stage salt tolerance; (3) to compare the results of screening the same population using either a hydroponic or soil screening method. Identification of salt-tolerance genotype(s) and genes with novel source and their use in breeding program could provide a breakthrough in breeding for salinity tolerance in rice.

## Materials and Methods

### Plant Materials

The Bengal and Assam Aus Panel (BAAP) comprises of 300 cultivars (of which 266 are *aus*) with 2 million SNPs (full details in Norton et al., 2018). A total of 204 cultivars from BAAP were randomly selected and used in this study, 187 of these are *aus* rice and 184 have SNPs data for GWAS (Supplementary Table S1). The BAAP also includes a number of improved cultivars one of which is the *indica* cultivar BRRI dhan 47, produced at the Bangladesh Rice Research Institute specifically to be salt tolerant (Mondal et al., 2019). Additionally, seven checks non*-aus* cultivars were used; two accessions of Pokkali (Bonilla et al., 2002) and one Hor Kuch (Noor et al., 2019) were tolerant checks, while IR 64 (Nakhoda et al., 2012), IR 36 (Rao et al., 2013), IR 29 (Bonilla et al., 2002), and Italica Carolina were susceptible checks. One Pokkali is in the BAAP (written POKKALI here) and the other was sourced directly from the International Rice Research Institute (written Pokkali) as was IR 29 and IR 64. IR 36 and Italica Carolina were sourced from the Rice Diversity Panel 1 (Zhao et al., 2011) and Hor Kuch from Dhaka University.

### Evaluation of Salt Tolerance at the Seedling Stage in Hydroponic Systems

Seeds were surface sterilized in 1% sodium hypochlorite followed by washing with deionized water and germinated for 3 days at 30°C. Five germinated seeds from each cultivar were selected and grown in Yoshida’s nutrient solution (Yoshida et al., 1976) in boxes (66 × 42 × 30 cm). Each box contained a 61 cm × 39 cm foam polystyrene float with 112 holes and a mesh was fixed to the plate to maintain the seeds. Each polystyrene plate had 102 cultivars of BAAP and five check cultivars. After 1 week in Yoshida’s solution, two uniform seedlings of each cultivar were selected and grown in control and salinity stress boxes separately. The new polystyrene plates had a small soft bung to hold the seedlings was used (Supplementary Figure S1). Each replicate run consisted of four boxes, two for control and another two for salt treatment. For the control, the seedlings were grown in Yoshida’s solution for 35 days. For the salt treatment, the salinity with strength of 1.5 g L^–1^ NaCl (∼4 dS m^–1^) was applied on 15th day and the treatment strength increased to 3 g L^–1^ NaCl (∼7 dS m^–1^) on 21st day and this salinity strength was maintained for the next 2 weeks till harvesting. The volume of Yoshida’s solution in each box was 40 L in the first 4 weeks while increased to 50 L in the last week. The Yoshida’s solution was changed every week during the experiment and the pH value was adjusted to 5.5 every 2 days by adding HCl or KOH solution. The experiment was conducted in the greenhouse in 2018 with 25°C minimum temperature, with 12 h day^–1^ supplementary light of 150 μM m^–2^ s^–1^ PAR. The experiment consisted of six replicate runs, the first and second replications being conducted from 5th May to 12th June, a third replication from 7th July to 13rd August, a fourth replication from 14th July to 21st August, a fifth replication from 16th July to 23rd August, and a sixth replication from 22nd July to 29th August.

### Evaluation of Salt Tolerance at the Seedling Stage in Soil Systems

Seeds were surface sterilized in 1% sodium hypochlorite and germinated at 30°C for 3 days. Then two uniform germinated seeds per cultivar were selected and grown in two boxes (control and salt treatment) with dimensions; 100 cm long, 70 cm wide and 28 cm deep each filled with 140 L of commercial topsoil (Don Valley Ltd, Inverurie, United Kingdom). Using a grid system of 18 rows and 12 columns, two germinating seeds were sown every 5 cm using a template as a guide (Supplementary Figure S2A). Surrounding all the test cultivars was an external border of the check varieties Pokkali and IR 64 using the same plant spacing to prevent edge effects. After 1 week, the seedlings were thinned from two plants to one, and the template removed (Supplementary Figure S2B). Plants were irrigated two times every day (morning and evening) to keep the soil moisture in the first and second weeks, growing initially aerobically. After 2 weeks of aerobic growth, the boxes were flooded initially to 3 cm above the soil and then maintained with a water level between 1 and 5 cm above the soil. In the control box, tap water was used. In the salt treatment box 3.0 g L^–1^ NaCl (∼ 6 dS m^–1^) in tap water was used. The plants were harvested after 5 weeks growing in soil, being 3 weeks with salt stress. The first replication was conducted from 4th May to 11st June, a second replication from 11st May to 18th June, a third replication from 28th May to 5th July, and a fourth replication from 4th June to 12nd July 2019. In the third and fourth replicates six rhizon samplers were buried horizontally at 5 and 15 cm depth in soil in each box to allow sampling of pore water to measure the electricity conductivity (EC) of the soil pore water. This soil experiment was conducted in the same greenhouse as the hydroponics experiment.

### Physiological Traits Evaluated

Salt injury score (SIS) was recorded according to the 1–9 scale as described in the Standard Evaluation System for salinity tolerance (Gregoria et al., 1997). Tiller number was counted the day before harvesting. After harvest shoots and roots were washed with tap water and then rinsed with deionized water three times. The shoot length (SL) and root length (RL) were measured after washing (RL was not assessed in the soil experiment). The shoot and root samples were collected separately and dried in an oven at 65°C for 4 days, after which shoot biomass (SB) and root biomass (RB) were weighed.

### Shoot Sodium and Potassium Content Analysis

Initial experiments were conducted to validate pooling of replicates for sodium (Na) and potassium (K) was assessed. Whole shoots dry mass of all replicates of 35 cultivars (20 cultivars from hydroponic experiment and 15 cultivars from the soil experiment) including the check cultivars plus randomly selected BAAP cultivars grown in salt stress condition (total number of samples 215) were cut into small pieces. Approximately 0.1 g dry matter of each replicate was taken and mixed into a pool for each cultivar. For each cultivar, both individual replicate and the pooled samples were digested using nitric acid and hydrogen peroxide on a block digester as described in Allen et al. (1974). After digestion, the samples were analyzed by Microwave Plasma Atomic Emission Spectroscopy (MP—AES) (Agilent 4100) to determine the Na and K concentration. The range of calibration standards for Na and K were 0.5–5 and 1.0–12.5 mg L^−1^, respectively, with 6 calibration points. A comparison for the selected cultivars of the mean element composition averaged from the replicates and that of a single pooled sample comprising equal weights of those replicates revealed significant correlations for Na and K content and the Na/K ratio (Supplementary Figure S3). Therefore, pooled samples for each cultivar were used to analyze the Na and K content in the BAAP population. This was done by preforming the pooled analyze protocol described above on all cultivars in the salt treatments for the hydroponic experiment and soil experiment.

### Data Analysis

ANOVA and correlation analysis were performed using Minitab 19 and R version 3.6.3. Relative root biomass (RRB), relative shoot biomass (RSB), relative root length (RRL), and relative shoot length (RSL) between the salt stress and control were calculated using the following equation:

$$\begin{array}{c} R\left(\%\right)=T/C\times 100\% \end{array}$$

where R is the relative value of the traits, and T and C are the trait values in the treated and control conditions.

Principal component analysis for traits were performed using the package “factoextra” in R version 3.6.3. To identify the salt tolerant cultivars in hydroponic and soil separately, cultivars were first ranked based on both the SIS and RSB, then the rankings of the two traits were added together., The cultivars were also categorized based on the Na/K ratio in hydroponic and soil systems.

### Genome-Wide Association Mapping

The relative value of growth traits, SIS, and ion content were used for running GWA mapping. GWA mapping was performed on 184 *aus* cultivars using PIQUE (Parallel Identification of QTL’s Using EMMAX7) to pre-process genotype and phenotype followed by EMMAX analyses on each phenotype in parallel (GitHub repo for PIQUE) as described in Norton et al. (2018). SNPs with minor allele frequency (MAF) < 0.05 were filtered out and maximum per-SNP missing was set at 5% for GWA. A mixed effects model was used to estimate the association between each SNP and phenotype across all cultivars. For the fixed effects, population structure was included as covariates based on the first five principal components of PCA. Random effects were estimated using a kinship matrix to measure the genetic similarity. The false discovery rate (FDR) of detected associations was estimated to calculate Benjamini–Hochberg adjusted probabilities (Benjamini and Hochberg, 1995). A significance threshold of 10% FDR was used to identify putative SNP associations (McCouch et al., 2016). Detail information see the Norton et al. (2018).

After GWA, the significance threshold for association between SNP and traits was set at *P* < 0.0001, a value previously used for this population (Norton et al., 2018, 2019). CLUMP analysis was conducted to identify multiple significant SNPs (*p* < 0.0001) that represent a single QTL using PLINK (Purcell et al., 2007) with parameters “–clump-p1 0.0001 –clump-p2 0.0001 –clump-r2 0.3 –clump-kb 243” as described in Norton et al. (2019) However, QTLs were discarded if the total number of significant SNPs (*p* < 0.0001) in a “clump” was less than 2. Clump analysis was done for each trait separately. Local linkage disequilibrium (LD) decay was estimated as r^2^, r^2^ values in each SNP pair in each region 500 kb upstream and downstream were calculated and visualized as a local Manhattan plot against a LD heatmap using the LDheatmap package in R version 3.6.3.

### Identification of Candidate Genes

For each QTL, a genomic distance of 243 kb (the global LD decay of the population; Norton et al., 2018) around the peak SNP call was defined and annotation of all genes in the regions were obtained from Rice Genome Annotation Project (RGAP)^1^, release 7. For subsequent analysis, those annotated as “(retro) transposon,” “hypothetical,” or “unknown” were excluded from the analysis. To identify which of these genes were good candidates for resistance to salt, we further examined these genes’ information such as gene ontology classification in RGAP, which provide insight into genes function and known genes orthology/homology, and compared the positions of these genes (QTLs) with genes previously reported to be involved into salt stress in rice.

### Assignment of Genotypes Clusters Within Candidate Regions

The Neighbor-Joining (NJ) method was used to identify cultivars that share a similar genetic background within the QTL. The SNPs used to generate the NJ tree were all the SNPs in each QTL identified by CLUMP. The NJ tree was bootstrapped 1,000 times using MEGA X (Kumar et al., 2018) and clusters of genotypes were identified at a bootstrap value of 95%. Once the cultivars were assigned to a cluster then the phenotypic response for the cultivars with each cluster were observed.

## Results

### Growth Characteristics in Hydroponic and Soil Systems

**FIGURE 1:**
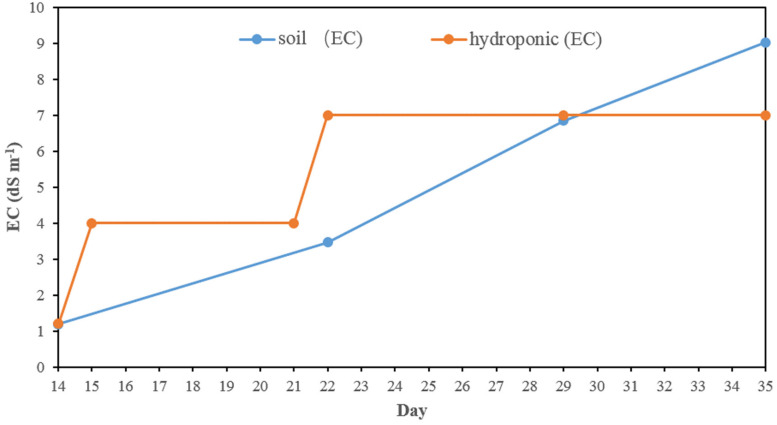
The electrical conductivity in salt stress in hydroponic and soil systems.

In hydroponics, the electricity conductivity (EC) of normal Yoshida’s solution was 1.2 dS m^–1^ in the first 2 weeks (Figure 1). The EC was ∼4 dS m^–1^ from day 15 after half salt stress applied and was increased to ∼7 dS m^–1^ from day 21, where it was kept until harvesting by changing the solution weekly. The EC in the soil experiment slowly rose from 1.2 to 9.0 dS m^–1^ after the salt stress was applied.

**FIGURE 2:**
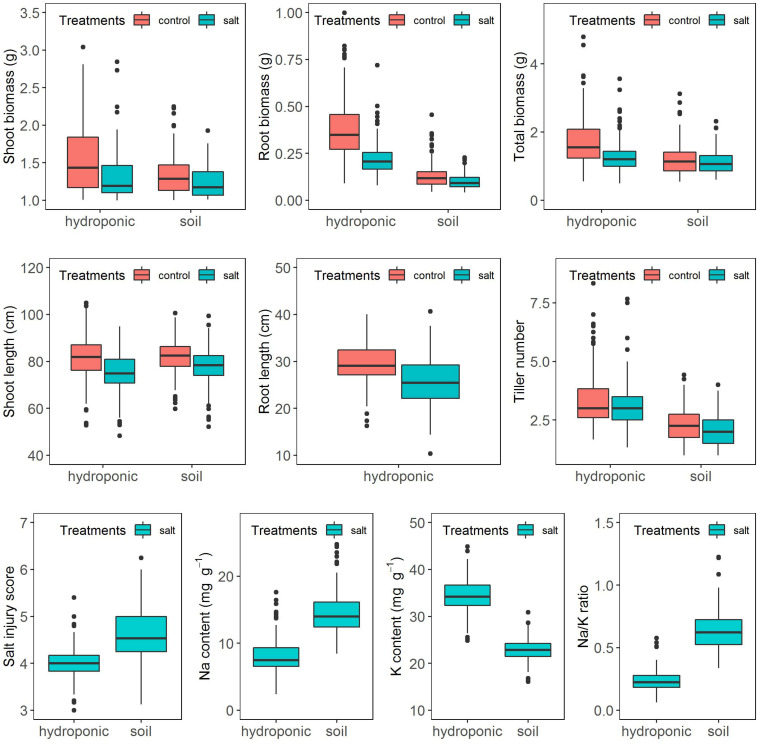
Response of the BAAP cultivars in growth traits, salt injury score and ion content when grown in the hydroponic and soil systems.

The salinity treatment negatively and significantly affected all investigated growth traits (Figure 2 and Supplementary Table S2) in both systems. For instance, under control, the average shoot biomass was 1.36 and 1.07 g in hydroponic and soil system, respectively, while under salt stress, it decreased to 1.04 and 0.98 g, respectively. Three-way ANOVA on plant growth traits with factors genotype, treatment and screening system showed there were significant system effects (proportion of variation ranged from 0.5 to 24.3%), treatment effects (0.83–10.6%) and genotypes effects (20.6–54.9%) in all traits (Table 1). Significant system × treatment interactions (0.02–3.3%), system × genotype interactions (5.9–9.6%), treatment × genotype interactions (3.4–6.5%) and system × treatment × genotype interactions were also observed in all growth traits except for the root biomass. The contribution of these interaction terms to the variation was small compared to the genotype contribution indicating that genotypic variation was the major factor in the observed phenotypic variation for the investigated traits.

**TABLE 1:** Percentage contribution of system, treatment and genotype factors to the observed variation of growth traits by three-way ANOVA.

Factor/interaction	Traits				
	Shoot biomass	Root biomass	Total biomass	Shoot length	Tiller number
System (S)	2.6%***	24.3%***	5.6%***	0.5%***	18.0%***
Treatment (T)	4.1%***	10.6%***	6.0%***	7.0%***	0.83%***
Genotype (G)	46.2%***	20.6%***	42.6%***	54.9%***	38.9%***
S × T	1.1%***	3.3%***	1.7%***	0.4%***	0.02%*
S × G	9.6%***	5.9%***	8.1%***	6.6%***	7.1%***
T × G	6.0%***	5.4%***	6.5%***	3.9%***	3.4%***
S × T × G	3.1%***	2.20%	3.1%****	2.1%**	2.8%***

*p < 0.05, **p < 0.01, ***p < 0.001.

For each measured growth traits, taking in to account the intrinsic difference in growth rate between accessions, the relative value of each trait was used as the indices of response to stress. There were significance genotypic differences for the salt stress response indices, with genotype differences explaining 5.2–55.2% of the variation in hydroponic system, and 7.1–62.3% in soil system (Supplementary Table S3).

### Na, K Content, and Na/K Ratio in Shoots in Hydroponic and Soil Systems

Ion content was assessed in six replicates of 20 selected cultivars in the hydroponics and in four replicates of 15 selected cultivars in the soil system. One-way ANOVA showed that there were significant genotypic variations in Na content (Hydroponic: *R*^2^ = 32.8%, Soil: *R*^2^ = 44.5%), K content (Hydroponic: *R*^2^ = 28.8%, Soil: *R*^2^ = 49.5%) and Na/K ratio (Hydroponic: *R*^2^ = 30.2%, Soil: *R*^2^ = 51.2%) (Supplementary Table S4). A comparison for the above selected cultivars of the mean element composition averaged from the replicates and that of a single pooled sample comprising equal weights of those six or four replicates revealed significant correlation for Na and K content and the Na/K ratio (Hydroponic: Na, *r* = 0.77; K, *r* = 0.65; Na/K, *r* = 0.75. Soil: Na, *r* = 0.83; K, *r* = 0.62; Na/K, *r* = 0.83) (Supplementary Figure S3). Moreover, the tolerance check (Pokkali) had the lowest Na content and Na/K ratio while the sensitive checks (IR29 or IR 36) had relative high Na content and Na/K ratio in both pooled and replicated treatments of both hydroponic and soil systems (Supplementary Figure S3) as expected. Thus, the pool sample of each cultivar were used to analyze the Na and K content of BAAP population. The frequency distribution of Na, K content, and Na/K ratio in BAAP population are presented in Supplementary Figure S4. The average Na and K content under salt stress condition in BAAP population were 8.0 and 34.3 mg g^–1^, respectively, in hydroponic system, and 14.5 and 22.9 mg g^–1^ in soil system (Figure 2 and Supplementary Table S2). The Na/K ratio across the BAAP population was 0.24 in hydroponic and 0.64 in soil system. These results showed that the plants in the soil system take up more Na than in the hydroponic system.

### Relationship Between Hydroponic and Soil Systems

The correlations between growth traits (shoot biomass, root biomass, shoot length, root length, and tiller number) were significant and positive under control and salt treatments in hydroponics (r ranged from 0.483 to 0.995 and 0.269 to 0.996, respectively) (Supplementary Table S5) and in soil (r ranged from 0.288 to 0.967 and 0.214 to 0.973, respectively) (Supplementary Table S6). Under salt stress, most of these growth traits were found to be significantly and negatively correlated with Na content and SIS while positively correlated with K content in both environments although that relationship was stronger in hydroponics than in soil. The negative correlation between Na and K content was significant in hydroponic but was not significant in soil.

**FIGURE 3:**
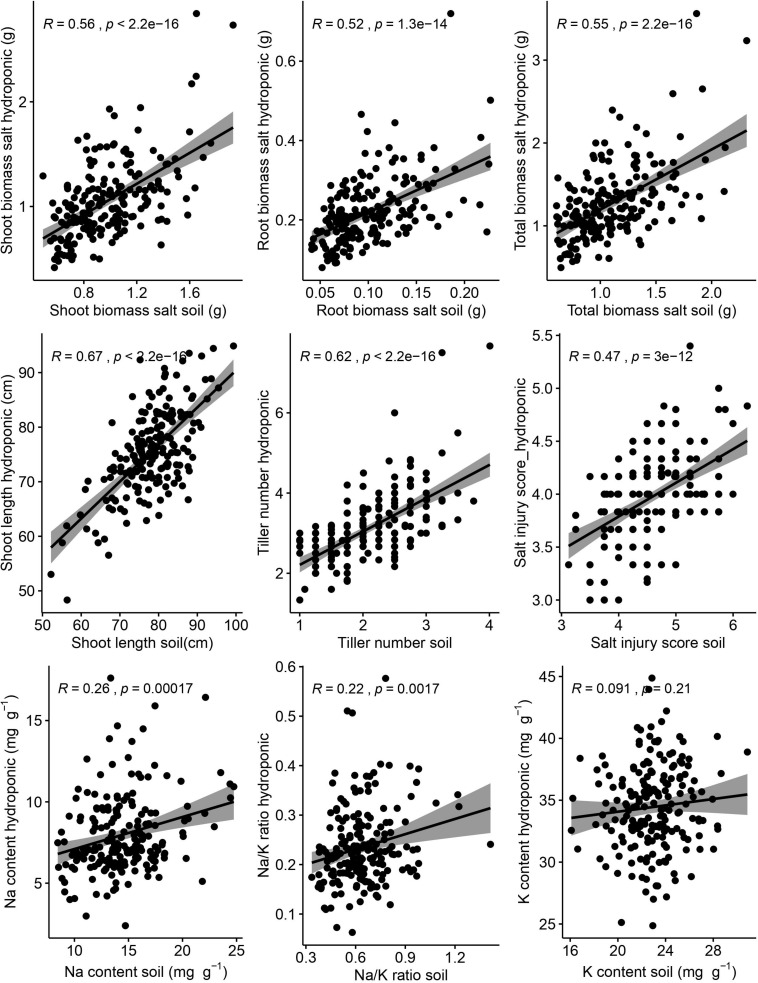
Relationship between hydroponic and soil environments under salt stress of corresponding traits. Salt injury score was analyzed by Spearman correlation and other traits was analyzed by Pearson correlation. The shaded area represents 95% confidence intervals.

Correlations between hydroponic and soil environments under salt stress of corresponding traits were performed (Figure 3). There were significant and positive correlations between hydroponic and soil systems of corresponding traits (shoot length, *r* = 0.67, *p* < 0.001; tiller number, *r* = 0.62, *p* < 0.001; shoot biomass, *r* = 0.56, *p* < 0.001; root biomass, *r* = 0.52, *p* < 0.001; SIS, *r* = 0.47, *p* < 0.001; Na, *r* = 0.26, *p* < 0.001; Na/K ratio, *r* = 0.22, *p* = 0.002). But the correction of K content was week between hydroponic and soil systems and was not significant.

The principal components (PC) analysis indicated that PC1 and PC2 explained 65.0 and 61.5% of the total variation for the salt related traits from the hydroponic and soil system, respectively (Supplementary Figure S5). In the PC biplot, the RSB, RRB and relative total biomass were grouped together, and the Na content and Na/K ratio were grouped in another group with similar directionality to SIS.

### Identification Salt Tolerant Cultivars According to Traits Performance in Hydroponic and Soil Systems

**FIGURE 4:**
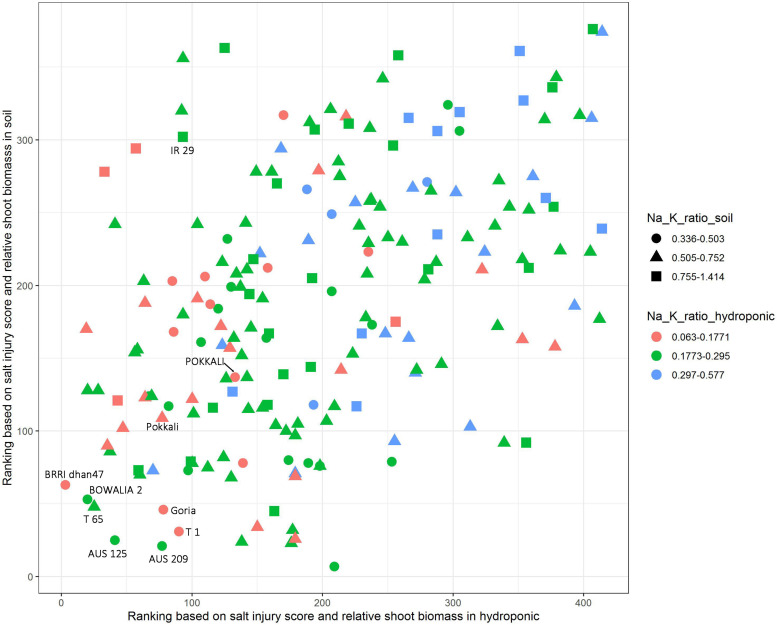
Identification of salt tolerant genotypes based on salt injury score, relative shoot biomass and Na/K ratio in hydroponic and soil systems. The cultivars were ranked based on combined salt injury score and relative shoot biomass in hydroponic and soil system, separately. In the hydroponic the Na/K ratio for the lowest 20% was 0.063–0.177 while in soil for the lowest 20% of the cultivars it was 0.336–0.503.

The salt jury score, RSB and Na/K ratio were used to identify the salt tolerant cultivars. The cultivars were first ranked based on combined SIS and RSB in the hydroponic and soil system, separately (Figure 4). Additionally, to explore the Na/K ratio, the cultivars were split into three groups based on the Na/K ratio for each the experimental conditions, these were the lowest 20% Na/K ratio, the highest 20% Na/K ratio and the 60% that fell in the middle. In the hydroponic system, the Na/K ratio for the lowest 20% was 0.063–0.177 while in soil for the lowest 20% of the cultivars it was 0.336–0.503 (Figure 4). The salt tolerant check POKKALI (from the BAAP) had a low Na/K ratio in both systems while Pokkali (from IRRI) had a low ratio in hydroponics but not soil. Many cultivars, including BRRI dhan 47, BOWALIA 2, T 65, AUS 125, Goria, T 1, and AUS 209 performed better than the Pokkali check cultivars, in terms of the ranking of the SIS and RSB. When used all three traits as the criterion in measuring salt tolerance, three cultivars, BRRI dhan 47, Goria, and T 1, showed better overall performance than the Pokkali cultivars, having low Na/K ratio and SIS while high RSB and can be considered as highly salt tolerant cultivars.

### Salt Tolerance by *aus* Subpopulation

The BAAP population was divided into five distinct groups by analysis of the population structure (Norton et al., 2018). The phenotypic trait variations in hydropoic and soil systems in different fastSTRUCTURE groups are presented in the Supplementaty Figures S6, S7. Except for relative tiller number for the plants grown in soil and K concentration for the plants grown in soil, there was a signficant difference in trait values according to group as revealed by one-way ANOVA (*P* < 0.01 except K in soils- *P* = 0.022). Under both systems, the cultivars in groups 1, 4, and 5 had the lower values in Na content, Na/K ratio and SIS, and had higher values in relative growth traits. The results suggested these three groups are salt tolerant cultivars. The group 1 showed highly salt toleance in both systems, and the group 4 showed more salt tolerance than group 5 in the hydroponic system but less salt tolerance than group 5 in the soil system. Under both systems, the groups 2 and 3 had the higher values in Na content, Na/K ratio and SIS, and had low relative growth values (RSL and relative biomass) suggesting there are salt sensitive cultivars. But the group 2 showed more salt sensitivity than group 3 in hydroponic system and less salt sensitivity than group 3 in soil system.

### GWA Mapping in Hydroponic System

**FIGURE 5:**
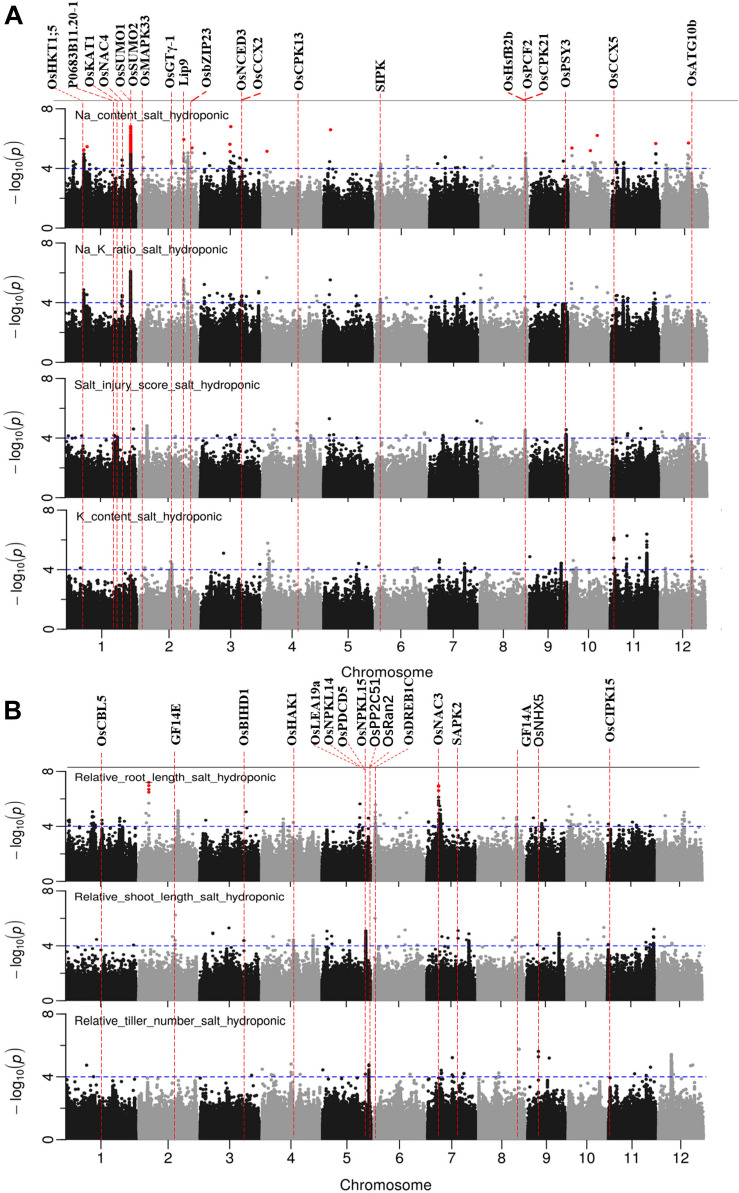
Manhattan plots for genome-wide association for salt related traits in hydroponic system. **(A)** Na content, Na/K ratio and salt injury score. **(B)** relative root length, relative shoot length and relative tiller number. Candidate genes detected within 243 kb of peak SNP regions known to be previously associated with salt tolerance are shown along on the top. The guide-line in blue is shown at –log10 (0.0001) = 4. Benjamini-Hochberg adjusted probabilities > 0.1 are highlighted with a red dot.

GWAS was conducted on 10 salt related traits for 184 *aus* cultivars of BAAP population (Figure 5 and Supplementary Figure S8). A total of 1,574 significant (*p* < 0.0001) SNPs were associated with at least one of these ten traits. CLUMP was used to identify multiple SNPs that represent a single QTL. Twenty QTLs were found to be significantly associated with Na content, 10 QTLs with K content, 16 QTLs with Na/K ratio, and 12 QTLs with SIS (Supplementary Table S7). The most notable QTL was observed on chromosome 1∼40 Mb associated with Na content and Na/K ratio with a total of 418 significant SNPs. Another notable QTL on chromosome 1 was found between 11.36 and 11.52 Mb associated with Na content and Na/K ratio with a total of 171 significant SNPs. Meanwhile, two QTLs were associated with both SIS and ion content. The QTL found on chromosome 8∼27.15 Mb was associated with SIS and Na content, while the QTL on chromosome 11, between 9.86 and 10.16 Mb, was associated with SIS, Na/K ratio, and K content (Table 2).

**TABLE 2:** Major QTLs detected for salt related traits in rice grown in hydroponic system.

		Position of peak SNP (Mb)									
Chr	QTL interval (Mb)	RSB	RRB	RTB	RSL	RRL	RTN	SIS	Na	Na/K	K
1	11.36–11.52								11.382^b^	11.382^a^	
1	34.94–34.94								34.937^a^	34.937^a^	
1	39.94–40.12								40.086^c^	40.067^c^	
1	41.85–41.85				41.85^a^			41.85^a^			
2	6.25–6.25					6.253^*d*^					
2	22.47–22.58				22.582^c^						
2	27.46–27.51								27.484^b^	27.484^b^	
2	29.99–30.05								30.046^b^	29.994^a^	
4	17.68–17.85	17.813^a^		17.813^a^			17.846^a^				
4	30.17–30.17	30.173^a^	30.173^a^	30.173^a^							
4	31.70–31.77	31.704^a^		31.704^a^	31.71^a^						
5	26.95–27.03				26.99^b^						
6	2.92–3.12								3.116^a^	3.116^a^	
6	19.50–19.58				19.579^b^				19.511^a^		
6	30.05–30.11		30.102^a^	30.106^a^							
7	7.24–7.57					7.239^c^					
7	8.30–8.58					8.368^b^	8.368^a^				
8	0.01–0.12	0.123^a^		0.123^a^						0.005^a^	
8	23.89–23.98					23.967^a^					
8	27.14–27.16							27.155^a^	27.155^b^		
9	19.87–19.90				19.876^a^						
9	22.01–22.03							22.033^a^			
10	0.58–0.58								0.58^b^	0.58^b^	
10	18.98–19.10	19.084^b^	19.096^b^	19.084^b^							
11	2.24–2.24										2.242^c^
11	7.21–7.39								7.372^a^	7.211^a^	
11	9.86–10.16							10.045^a^		10.161^a^	10.042^a^
11	22.43–22.60										22.489^c^
12	7.89–8.13			7.986^a^			8.016^b^				

*The range of the QTL is presented as the QTL interval, and the position of the most statistically significant SNP for the trait association is given. Letters indicate the level of statistical significance ^*a*^1 × 10^–5^ ≤ P-value < 1 × 10^–4^; ^*b*^1 × 10^–6^ ≤ P-value < 1 × 10^–5^; ^*c*^1 × 10^–7^ ≤ P-value < 1 × 10^–6^; P-value < 1 × 10^–7^.*

There were 23, 18, 4, 6, and 7 QTLs associated for RRL, RSL, RRB, and RSB, relative tiller number, respectively (Figure 5B and Supplementary Table S7). Two notable QTLs (in terms of statistical significance) were found on chromosome 2∼6.25 Mb, and chromosome 7∼7.23 Mb which were associated with RRL. A QTL on chromosome 10∼19.08 Mb was detected for RSB, RRB and relative total biomass with a total of 177 significant SNPs. A QTL located on chromosome 8 between 0.01 and 0.12 Mb was associated with RRB, RSB and Na/K ratio.

In total, 97 significant QTLs for 10 salt related traits in hydroponic system were identified (Supplementary Table S7). Out of these 97 QTLs, twenty QTLs were associated for multiple traits (Table 2). These QTLs were located; one QTL each on chromosomes 7 and 12, two each on chromosomes 2, 8, 10, and 11, three on chromosome 4, and four on chromosome 1.

### GWAS Mapping in Soil System

**FIGURE 6:**
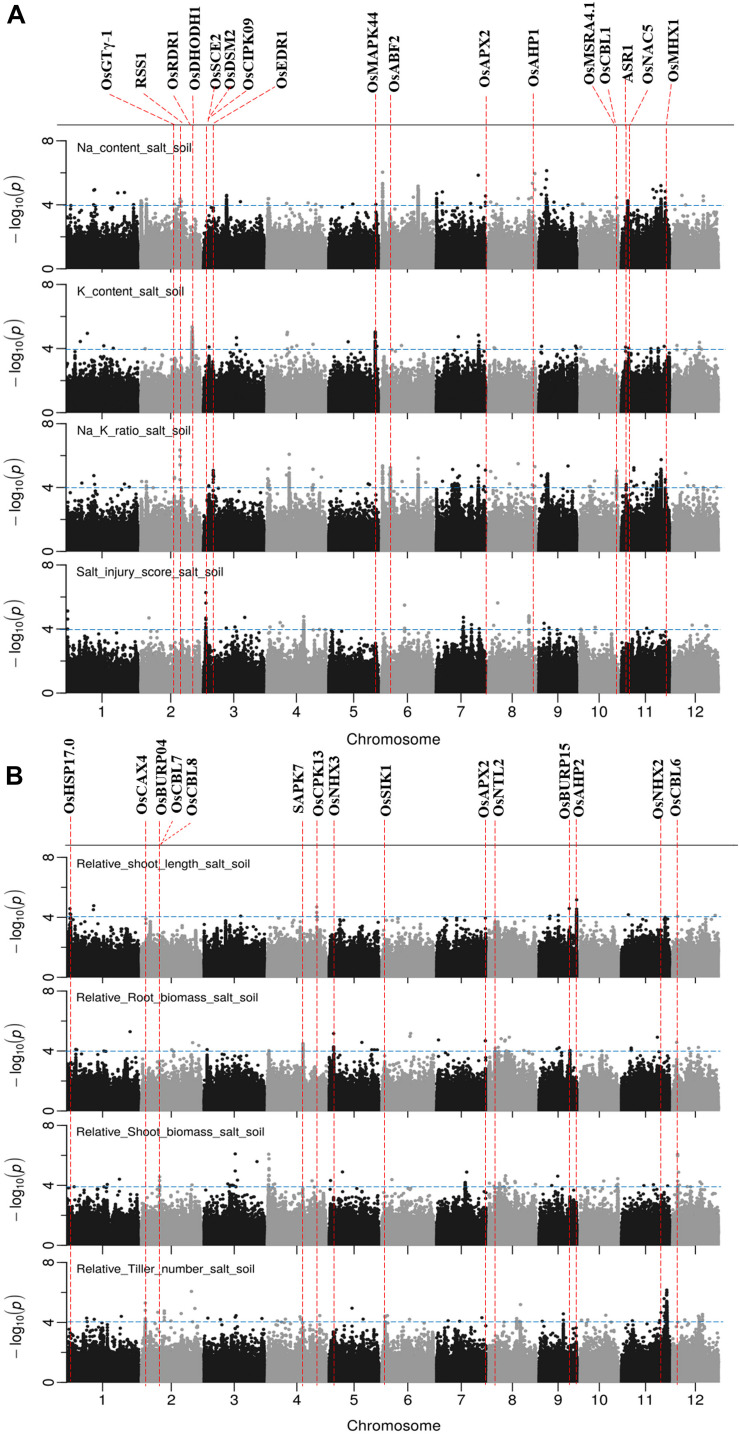
Manhattan plots for genome-wide association for salt related traits in soil system. **(A)** Na content, Na/K ratio and salt injury score. **(B)** relative root length, relative root biomass, relative shoot biomass, and relative tiller numbers. Candidate genes detected within 243 kb of peak SNP regions known to be previously associated with salt tolerance are shown along on the top. The guide-line in blue is shown at –log10 (0.0001) = 4.

The results of GWAS mapping in soil system are presented in Figure 6 and Supplementary Figure S9. A total of 2,054 significant (*p* < 0.0001) SNPs were associated with at least one of the nine traits investigated in soil system and CLUMP analysis were used to group them into QTLs. There were 20, 4, 26, and 8 QTLs associated with Na content, K content, Na/K ratio and SIS, respectively (Figure 6A and Supplementary Table S8). Two QTLs detected for Na and Na/K ratio were notable (in terms of statistical significance), one located on chromosome 6∼0.67 Mb with a total of 75 significant SNPs, and the other one was on chromosome 6∼22.03 Mb with a total of 350 significant SNPs. Besides these QTLs, two QTLs detected for K content need to be noted, one on chromosome 2∼30.80 Mb and the other one on chromosome 5∼27.90 Mb. The notable QTL associated with SIS was located on chromosome 3∼1.42 Mb (Table 3).

**TABLE 3:** Major QTLs detected for salt related traits in rice grown in soil system.

		Position of peak SNP (Mb) and *P*-value								
Ch	QTL interval (Mb)	RSB	RRB	RTB	RSL	RTN	SIS	Na	Na/K	K
2	3.31–3.43							3.341^a^	3.361^a^	
2	23.52–23.60							23.519^a^	23.519^c^	
2	30.78–31.05									30.804^b^
3	1.40–1.46						1.42^c^			
3	18.79–18.79	18.791^c^		18.791^a^						
4	0.49–0.78							0.78^a^	0.592^b^	
4	0.73–0.96	0.957^b^								
4	13.35–13.44								13.442^c^	
5	2.64–2.66		2.644^b^	2.663^a^						
5	27.90–27.91									27.903^b^
6	0.66–0.79							0.672^c^	0.667^b^	
6	5.20–5.43								5.375^b^	
6	21.90–22.17							22.027^b^	22.072^b^	
8	3.63–3.83		3.829^a^	3.629^a^						
8	24.29–24.31						24.304^a^			
8	27.82–27.85							27.82^b^	27.82^b^	
9	4.48–4.76							4.595^b^	4.659^a^	
9	5.14–5.39								5.372^a^	
9	22.60–22.64				22.628^b^					
10	22.04–22.22							22.057^a^	22.057^b^	
11	21.10–21.20							21.132^a^	21.132^a^	
11	23.79–23.99			23.795^a^				23.832^b^	23.976^b^	
11	27.08–27.45					27.253^a^				
12	3.07–3.07	3.066^c^		3.066^b^						

*The range of the QTL is presented as the QTL interval, and the position of the most statistically significant SNP for the trait association is given. Letters indicate the level of statistical significance ^*a*^1 × 10^–5^ ≤ P-value < 1 × 10^–4^; ^*b*^1 × 10^–6^ ≤ P-value < 1 × 10^–5^; ^*c*^1 × 10^–7^ ≤ P-value < 1 × 10^–6^; P-value < 1 × 10^–7^.*

There were 7, 6, 5, 3, and 10 QTLs associated for RSB, RRB, relative total biomass, RSL and relative tiller number, respectively (Figure 6B and Supplementary Table S8). Among these QTLs, three notable QTLs were found, one was on chromosome 11∼27.25 Mb containing 180 significant SNPs associated with relative tiller number (Figure 6B). The others were QTL on chromosome 4 ∼ 0.96 Mb associated with RSB and the QTL on chromosome 9∼22.63 Mb associated with RSL. It is noted that the QTL on chromosome 11 from 23.79 to 23.99 Mb identified for relative total biomass was also associated with Na content and Na/K ratio. In total, 74 QTLs were significantly associated with the nine salt related traits investigated in the soil system and out of them 14 QTLs were associated with more than one trait (Table 3). These QTLs were located: one on each of the chromosomes 3, 4, 5, 9, 10, and 12, two on chromosomes 2, 6, 8, and 11.

### Combing all QTLs in Hydroponic and Soil Systems

**FIGURE 7:**
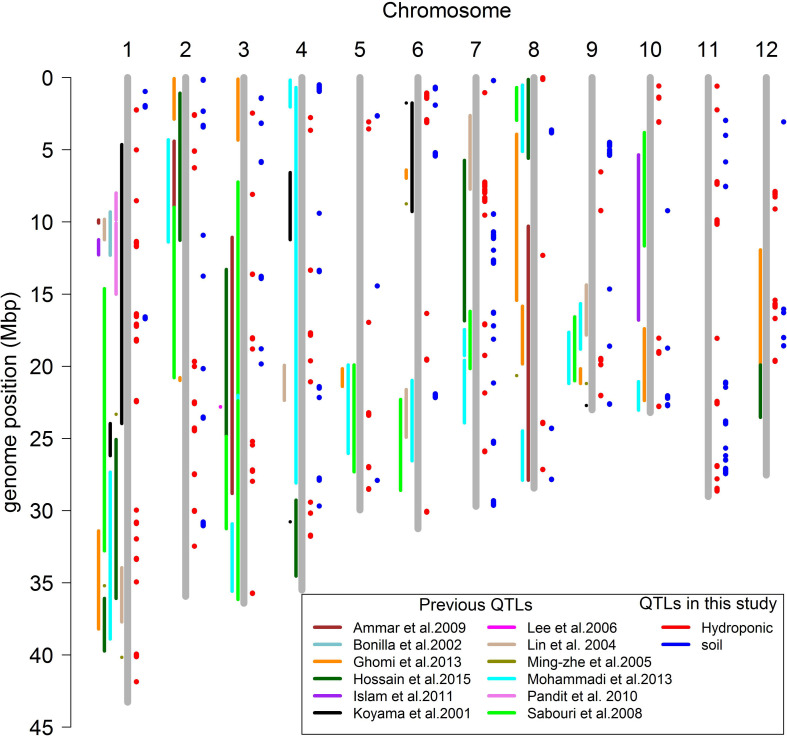
Comparison the positions of QTLs mapping for salt related traits in different studies.

A total of 160 QTLs were detected in the soil and hydroponic system for all salt related traits (Figure 7), and 48 of these QTLs were associated with multiple traits and/or systems, in which 11 QTLs were identified in both soil and hydroponic systems. These 11 QTLs were located; one on chromosomes 1, 2, 10, 11, 12 and two on chromosomes 3, 4, and 7 (Figure 7). A comparison of all 160 QTLs detected in hydroponic and soil systems with the previously published QTLs using bi-parental linkage mapping populations for salt tolerance identified that 94 of QTLs identified in this study were co-located with the previously published QTLs (Figure 7).

A total of 65 candidate genes were found in 52 QTLs (Figures 5, 6). The functional evidence that these candidate genes are involved into salt stress is presented in Supplementary Table S9. Notably, the well-known Na transporter gene *OsHKT1;5* was found to be in the QTL region on chromosome 1 between 11.35 and 11.52 Mb. Two post-translational modification_SUMOylation genes (*OsSUMO1* and *OsSUMO2*) were found in the most significant QTL region on chromosome 1∼40.00 Mb. Two genes *OsGTγ-1*, and *OsCPK13* previously reported to be involved in salt-stress in rice co-localized with QTLs detected in both systems. *OsGTγ-1* was detected in the QTL region located on chromosome 2∼20.0 Mb and the *OsCPK13* was found in the QTL region on chromosome 4 between 29.42 and 29.68 Mb.

### Chromosome 1: 39.94–40.12 Mb

**FIGURE 8:**
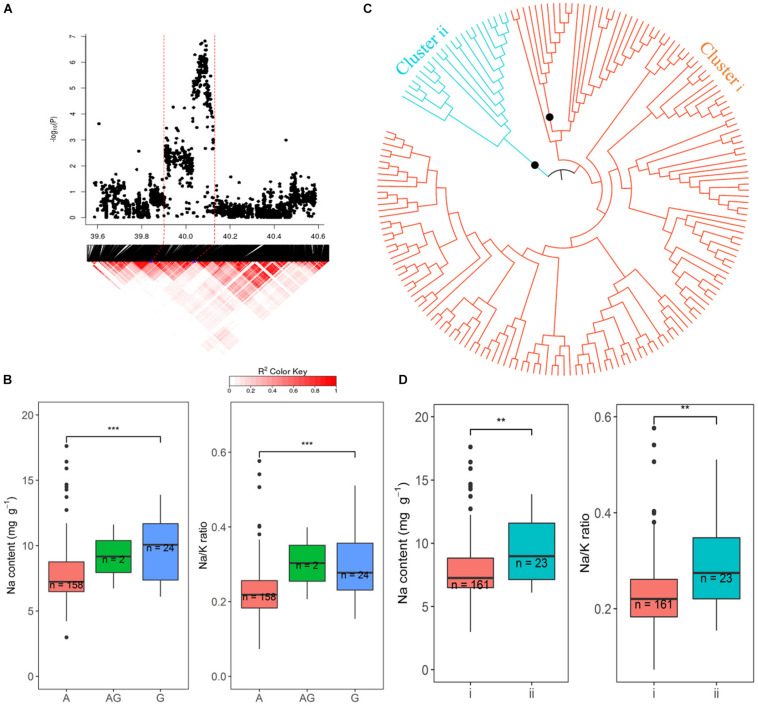
Significant associations for Na content and Na/K ratio on rice chromosome 1 between 39.94 and 40.12 Mb. **(A)** Local Manhattan plot (top) and LD heatmap (bottom). **(B)** Range of Na content and Na/K ratio variation observed for each allele for peak SNP 1:40086153. **(C)** Neighbor-joining tree for the BAAP cultivars for all SNP located between 39.94 and 40.12 Mb on chromosome 1, black circles indicted node support of ≥ 95%. **(D)** Range of phenotypic variation observed for each cluster in associated traits.

The promising QTL on chromosome 1∼40 Mb was further explored because of the number of SNPs and the significance of their association with Na and Na/K in hydroponics (Figure 8). There were 218 and 200 significant SNPs associated with Na content and Na/K ratios, respectively, from hydroponics. From the peak SNP (1:40086153) to ± 0.5 Mb, local LD decay was calculated to be approximately 130 kb, indicating that SNPs between 39.94 and 40.21 Mb probably represent a single QTL. The most statistically significantly associated SNP (1:40086153) in this QTL included three alleles, the homozygous A (*n* = 158), homozygous G (*n* = 24), and heterozygous AG (*n* = 2). The variation between the alleles (A and G) for Na content and Na/K ratio were significant. Cultivars with the A allele had lower Na content (average 7.8 mg g^–1^) and Na/K values (0.23) than cultivars with the G allele (average 9.5 mg g^–1^ and 0.29, respectively) (Figure 8B). All SNPs within the range of 39.94–40. 12 Mb were used to determine the genotypes clusters. Cluster analysis of SNPs data revealed two clusters (Figure 8C) which were identified at a bootstrap support value 95%, with 161 cultivars in cluster i and 23 cultivars in the cluster ii. The cluster i had lower Na content (average 7.9 mg g^–1^) and Na/K ratio (average 0.23) than that in cluster ii (9.2 mg g^–1^ and 0.28, respectively) (Figure 8D). The 158 cultivars with allele A for the peak SNP had low Na content were all in the cluster i, while the 22 of 24 cultivars with allele G for the peak SNP had high Na content were in the cluster ii. The salt tolerant cultivars, BOWALIA 2, T 65, AUS 125, Goria, T 1, and AUS 209 were in the cluster i (Supplementary Table S1).

### Haplotypes Analysis of Candidate Genes *OsSUMO1, OsSUMO2*, and *OsHKT1;5*

The haplotypes of two candidate genes (*OsSUMO1* and *OsSUMO2*) which were found in promising QTL on chromosome 1∼40 Mb region were determined. Three SNPs were found in the *OsSUMO2* and one SNP found in the *OsSUMO1*. There are three haplotypes in BAAP cultivars, CTT (*n* = 142), YTT (25) and TCC (17) for the *OsSUMO2*, and the SNP in the *OsSUMO1* was a G/C polymorphism with 157 cultivars having G, 25 cultivars having C and 2 cultivars were heterozygous. The average Na content in the cultivars carrying the haplotype CTT for *OsSUMO2* (7.84 mg g^–1^), and G for *OsSUMO1* (7.80 mg g^–1^) were significantly (*p* < 0.01) lower than the cultivars carrying for TCC for *OsSUMO2* (9.40 mg g^–1^), and C for *OsSUMO1* (9.40 mg g^–1^), while the average Na content was not significantly different in cultivars carrying the haplotype CTT and YTT for *OsSUMO2* (Supplementary Figure S10). Except for AUS 209 having the haplotype (YTT) for the *OsSUMO2*, the other salt tolerant cultivars Pokkali, BRRI dhan 47, BOWALIA 2, T 65, AUS 125, Goria, and T 1 had the low Na CTT haplotype for the *OsSUMO2* and G for *OsSUMO1* (Supplementary Table S1).

The haplotypes of candidate genes *OsHKT1;5* which was found in the QTL on chromosome 1∼11.38 Mb were determined, revealing a total of 34 SNPs in this candidate gene region. Four haplotypes named Hap.A (5), Hap.B (117), Hap.C (27), and Hap.D (8) (Supplementary Table S1) had more than 5 cultivars. The Na content in these four haplotypes were significantly (*p* < 0.001) different, with the Hap.B having lowest Na content (7.47 mg g^–1^), and Hap.C and Hap.D had high Na content (9.80 and 9.13 mg g^–1^, respectively). The salt tolerant cultivars Pokkali, BRRI dhan 47, AUS 125, AUS 209, T 65, T 1, and Goria had the low Na haplotype Hap.B.

## Discussion

### Comparison of Methods of Assessing Seedling Stage Salt Tolerance

In this study, two methods (soil and hydroponic systems) were used to assess the seedling stage salt tolerance in BAAP population. In both the salt stress was applied after 2 weeks in normal conditions and kept for 3 weeks. In the hydroponic system, the electricity conductivity (EC) of solution was ∼4 dS m^–1^ in the first week after salt stress applied, and then increased to 7 dS m^–1^ on day 21 where it was maintained by changing the solution weekly. However, in soil system, the pore water EC level slowly rose from 1.2 to 9 dS m^–1^ due to the salt accumulated in the soil (Figure 1). Comparison of these two systems revealed the average Na content (14.45 mg g^–1^), Na/K ratio (0.69) and SIS (4.64) in shoots growing in soil system were higher than that in hydroponic system (8.02 mg g^–1^, 0.24 and 3.99, respectively). However, the reduction of growth traits by salt stress in hydroponic was more serious than that in soil environment. For instance, the salinity stress reduced shoot biomass across the population by 23.5 and 8.4%, root biomass by 42.1 and 23.1%, and shoot length by 8.0 and 4.6% in hydroponic and soil system, respectively (Figure 2 and Supplementary Table S2). Since the toxicity of Na in the soil increased gradual, the early growth was likely unaffected, so the impact on growth traits was small, but later when Na levels in soil overtook those achieved in hydroponics, the shoot Na and SIS were high. As the Na content is considered as a selection criterion for salt tolerance (Munns et al., 2006), we further checked the relationship between the Na content with other salt related traits (relative growth value, SIS and K content), and found that the correlation between the Na content in shoots with other salt related traits in hydroponic system was stronger than that in soil system (Supplementary Tables S5, S6). In both systems, the commonly tolerant check Pokkali (Bonilla et al., 2002) and sensitive check IR 29 (Bonilla et al., 2002) were significantly different, with Pokkali having the low Na, Na/K ratio, and SIS while the IR 29 had high Na, Na/K ratio, and SIS (Supplementary Figure S4). Three other analyses point to the observation that the hydroponics and soil systems are similar but not the same in terms of genotypic response to salt stress. First, the three-way ANVOA for plant growth traits presented in Table 1 shows that while there was significant interaction between screening system and both treatment and genotype together for any trait, it did not explain as much of the variation as the genotype by treatment interaction. Secondly, the PC biplots (Supplementary Figure S5) were remarkably similar for both screening systems in terms of the direction of trait vectors. Finally, comparison of traits by subpopulation (Supplementary Figures S6, S7) also indicates the same pattern of differentiation in hydroponics and soil (e.g., both identify group 1 as tolerant, and group 3 sensitive). These results indicate that both systems worked in evaluation salt tolerance.

### Salt Tolerant Genotypes

Na/K ratio and visual symptom rating have been used for identifying the salt tolerant cultivars in many studies (Gregoria et al., 1997; Frouin et al., 2018). Gregoria et al. (1997) reported that Na/K ratio is a good parameter in quantifying the degree of salinity tolerance for molecular genetic studies and visual symptom rating is adequate to determine the level of tolerance for breeding purposes. Yeo et al. (1990) suggested using overall performance to identify tolerant accessions for breeding programs. In this study, three traits (Na/K ratio, salt injury, and RSB) were used to identify the salt tolerant cultivars. Several salt tolerant cultivars were identified, in which BRRI dhan47, Goria, and T 1 were noticeable as they had low Na/K ratio level like the tolerance check Pokkali, while they had better performance than Pokkali in RSB. BRRI dhan47 (IRRI line IR63307-4B-4-3) is a salt-tolerant cultivar derived from a cross between a somaclonal variant of Pokkali (TCCP 266-2-49-B-B-3) and the breeding line IR51511-B-B-34-B (Mondal et al., 2019). The salt-tolerant cultivar Pokkali underwent cell culture to produce somaclonal variation with improved agronomic traits, resulting in a variant, TCCP 266-2-49-B-B-3, with desirable agronomic characteristics and retained salinity tolerance similar to Pokkali (Gregorio et al., 2002). It is vigorous in growth, semi-dwarf and lodging resistant, with white pericarp and improved cooking quality compared to Pokkali (Mondal et al., 2019). That BRRI dhan47 showed better performance than Pokkali in response to salt stress maybe due to the favorable genes from the somaclonal variant of Pokkali (TCCP 266-2-49-B-B-3) and from IR51511-B-B-34-B that were incorporated into the BRRI dhan47.

The mechanism of salt tolerance in Pokkali and the newly identified salt tolerant cultivars were compared. In hydroponic and soil systems, the two Pokkali accessions had high biomass in both control and salt treatment (roughly twice the size of the average biomass) while the other salt tolerant cultivars identified here had lower than average biomass. The salt tolerance of Pokkali has been linked to its big plant stature that results in dilution of Na concentration in the leaves (De Leon et al., 2017). Since the cultivars that were identified as being more tolerant that Pokkali, were small plants it seems they do not utilize dilution as a tolerance mechanism. Presumably they utilize a mechanism of Na compartmentation into cell vacuoles and/or Na exclusion from the transpiration stream and therefore keep Na concentration in shoots low. Further research is needed to confirm this mechanism.

### QTLs for Seedling Stage Salt Tolerance

In this study, a total of 160 QTLs were identified for all salt related traits in hydroponic and soil systems (Figure 7). The QTLs identified here were compared with previously reported QTLs identified using bi-parental linkage mapping populations, revealing that 97 previously reported QTLs co-located with the QTLs in this study. Some of these QTLs were particularly noteworthy since they were associated with multiple traits/environments, containing a large number of significant SNPs, and co-localized with previously reported QTLs. For example, the QTL located at 11.35–11.52 Mb on chromosome 1 was associated with Na content and Na/K ratio in hydroponic system which had 109 and 62 significant SNPs, respectively, and was reported in four previous studies (Koyama et al., 2001; Bonilla et al., 2002; Pandit et al., 2010; Islam et al., 2011). The QTL at 39.9–40.1 Mb on chromosome 1 associated with Na content and Na/K ratio in hydroponics with 418 significant SNPs was reported in the study of Ming-Zhe et al. (2005). The QTL at 13.34–13.45 Mb on chromosome 4 was detected in soil and hydroponic systems and was co-located with a QTL for number of panicles under salt stress reported by Mohammadi et al. (2013). These results indicate that these are stable QTLs and the underlying genes could be important for breeding salinity tolerance in breeding programs.

In the last 5 years there has been 12 studies using GWAS to identity QTLs for salt tolerance in rice, and a comparison of these studies (in terms of population, screening methods, markers) is presented in the Supplementary Table S10. Most of these studies used *indica* and/or *japonica* accessions as the mapping population and evaluated the salt tolerance in one screening system. For example, Frouin et al. (2018) used 235 temperate *japonica* rice and evaluated salt tolerance at seedling stage with salinity stress 50 mM NaCl (EC ∼ 6 dS m^–1^) in hydroponic system. Lekklar et al. (2019) used 104 *indica* rice and evaluated their salt tolerance at heading stage with water 150 mM NaCl solution 9 days in soil system. Three of these studies include a few *aus* cultivars in their research materials. Liu et al. (2019) evaluated the salt tolerance of 708 rice accessions but only 16 *aus* rice. Rohila et al. (2019) used 162 rice accessions with 30 *aus* rice and Patishtan et al. (2018) used 306 rice cultivars with 39 *aus* rice as the mapping populations. A comparison of the positions of QTLs detected in this study with the previously reported significant SNPs/QTLs associated for salt tolerance using GWA mapping is presented in the Supplementary Table S11. A total of 74 and 53 QTLs detected in the current study for hydroponic and soil systems, respectively, were within the windows of 200 kb of previously reported significant SNPs / QTLs. For example, of the 97 and 74 QTLs detected in hydroponics and soil in this study, 57 and 35, respectively, were reported by the study of Patishtan et al. (2018). However, there are still a number of QTLs for salt tolerance detected in this study (21 in hydroponics and 19 in soil), which used *aus* rice as the mapping population that have not been previously reported. For instance, the QTL on chromosome 11 between 9.85 and 10.61 Mb associated with SIS, Na content and Na/K ratio in hydroponic system, and the QTL on chromosome 7 between 17.06 and 17.21 Mb associated with Na/K ratio in the hydroponic system and with RSB in the soil system are novel. These notable QTLs may be specific for *aus*.

### Candidate Genes for Salt Tolerance

A total of 65 genes previously reported to be involved with salt stress in rice were found within the peak SNP ± 243 kb (the global LD decay of BAAP population) of QTLs identified in this study (Figures 5, 6 and Supplementary Table S9). Out of these 65 genes, 11 genes were related to Ca^2+^ signaling including protein kinases. These genes are *SAPK2, SAPK7, OsCPK13, OsCPK21, OsCBL1, OsCBL5, OsCBL6, OsCBL7, OsCBL8, OsCIPK09*, and *OsCIPK15*. *OsCPK13* was detected in the QTL of chromosome 4∼29.41 Mb associated with SIS in the hydroponics and RSL in soil system. Several studies have reported that the overexpression of this protein increases the plant tolerance to salt, cold and drought stress and it was suggested to be the best candidate gene for rice salt tolerance improvement within the Ca^2+^ -dependent protein kinases (CDPK) families (Saijo et al., 2000; Wan et al., 2007). Generally, a transient increase in cytosolic Ca^2+^ is the first step in the response cascades to numerous environmental stimuli, including salt stress (Bouché et al., 2005; DeFalco et al., 2010).

Ten of the candidate genes in QTL regions detected in this study are for ion homeostasis (Figures 5, 6 and Supplementary Table S9). These genes are *OsKAT1, OsHAK1, OsCCX2, OsCCX5, OsCAX4, OsMHX1, OsNHX5, OsNHX3, OsNHX2*, and *OsHKT1;5. OsHKT1;5* (LOC_Os01g20160) (positioned at 11.45 Mb) is in the QTL region located on chromosome 1 between 11.36 and 11.52 Mb which was associated with Na content and Na/K ratio in hydroponics. *OsHKT1;5*, is a major contributor to *Saltol* and involved in retrieval of Na^+^ from the xylem. It has been reported that the expression of *OsHKT1;5* was induced about 2.4-fold under 100 mM NaCl compared to control (Shohan et al., 2019). Allelic variation of this gene has been shown to underlie important differences between tolerant and sensitive cultivars (Ren et al., 2005). In a stress situation, transporters have a crucial role on the control of the cell homeostasis (Maathuis et al., 1996). Transporters controlling the distribution of K^+^ and Na^+^ in plants are considered as one of the key determinants of plant salt tolerance due to their capacity to maintain a high cytosolic K^+^/Na^+^ ratio (Maathuis et al., 1996; Negrão et al., 2011).

Post-translational modifications (PTMs) are important in the response to environmental changes in plants because they can rapidly regulate the cell proteome to respond quickly new cellular needs. An important mechanism of the PTMs in plant response to salt stress is the regulation of the Salt Overly Sensitive pathway (Negrão et al., 2011). In the most significant QTL region on chromosome 1∼40 Mb which was associated with Na content and Na/K ratio in hydroponics, two post-translational modifications_SUMOylation genes (*OsSUMO1* and *OsSUMO2*) are small ubiquitin-like proteins involved in stress response and adaptation (Supplementary Table S9). It has been reported that salt stress induces accumulation of SUMO1/2-conjugated proteins in Arabidopsis (Conti et al., 2008). *OsSUMO2* and *OsSUMO1* have been shown to be, respectively, up and downregulated in mRNA levels in response to salt stress in rice (Chaikam and Karlson, 2010). In additional, in the QTL region located on chromosome 3 between 1.40 and 1.46 Mb which associated with SIS in the soil system (Supplementary Table S9), gene *OsSCE2* is a E2 SUMO-conjugating enzyme involved in stress response and adaptation which was upregulated in response to salt stress in rice seedlings (Nigam et al., 2008). This suggested SUMOylation of these genes might play an important role in development of rice under salt stress.

Many transcription factors (TFs) have been shown to be involved in plant responses to stress conditions (Saibo et al., 2009). TFs of AP2/EREBP, NAC, bZIP, MYB, and WRKY subfamilies have been reported as related to rice responses to salinity (Hossain et al., 2010; Ding et al., 2014). Eleven of candidate genes in QTLs region identified in this study are TFs (Supplementary Table S9). There were *OsNAC4, OsNAC3, OsNTL2, OsNAC5, OsbZIP23, OsABF2, OsPCF2, RSS1, OsBIHD1, DREB1C*, and *OsGTγ-1. OsGTγ-1* was detected in the QTL region located on chromosome 4 between 20.01 and 20.16 Mb which was associated with Na content in hydroponics and Na/K ratio in soil system. O*sGTγ-1* is a typical member of the GTγ subfamily in rice encoding a protein containing a conserved tri-helix domain (Fang et al., 2010). Fang et al. (2010) reported the transcript level of *OsGTγ-1* was strongly up regulated by salt-stress, while the *OsGTγ-1* mutant is more sensitive to salinity and overexpression of the gene in rice improves salt-stress tolerance.

Two *aus* cultivars (T 1 and Goria) were identified as more salt tolerant than Pokkali. To determine if these cultivars have novel alleles for candidate genes, the haplotype analysis tool of RiceVarMap (Zhao et al., 2015), which included cultivars from the Rice Diversity Panel from which some of the BAAP were derived, was employed. Only the *aus* cultivar Goria and Pokkali are tolerant in the current study and in the RiceVarMap database (Supplementary Table S12). Haplotype analysis of *OsSUMO1* and *OsSUMO2* did not reveal alleles for Pokkali (*OsSUMO1)* or Goria (*OsSUMO1* and *OsSUMO2*), so it is not possible to determine if Goria has a different allele.

Seven major alleles of *OsHKT1;5* (*Japonica, Agami, Aus, Daw, IR 29, Hasawi, and Aromatic*) were previously identified in 39 *O. sativa* accessions based on *OsHKT1;5* gene sequence (Platten et al., 2013). Negrão et al. (2013) reported 17 haplotypes (A-Q) of *OsHKT1;5* based on 57 SNPs from 392 rice cultivars. Eleven cultivars were common to both studies. Nipponbare, Azucena, and Carolina Gold were in the allele group *Japonica* in the study of Platten et al. (2013) while they had different haplotypes [A (Nipponbare) and H (Azucena and Carolina Gold)] in the study of Negrão et al. (2013). Aswina and Hasawi were in the allele group *Hasawi* in Platten et al. (2013) and had different haplotypes (C and D, respectively) in Negrão et al. (2013). Pokkali (26869) and Nona Bokra had the *Aromatic* allele in Platten et al. (2013) and had haplotype E in Negrão et al. (2013). FL478, Rayada, and FR13A had the *Aus* alleles in Platten et al. (2013) had the haplotype H in Negrão et al. (2013). Examining the sequence of Goria generated in the current study using the Intergrated Genome Viewer (Thorvaldsdóttir et al., 2013) allowed comparison with 43 of the 57 SNPs in *OsHKT1;5* reported in the study of Negrão et al. (2013), revealing complete match only to haplotype E reported in the Negrão et al. (2013).

Goria, Pokkali and two Nona Bokra accessions are present in the RiceVarMap database (Zhao et al., 2015). Using 92 SNP markers only in the gene (not promoter or downstream) reveals three haplotypes for these accessions (out of 19 haplotypes in the whole database (Supplementary Table S12), with Goria identical to one Nona Bokra (IRS_313-7736) but differing by 12 and 13 SNPs, respectively, from the other Nona Bokra (CX273) and Pokkali which are themselves different in only one SNP. Together these haplotype analyses suggest that Goria has an allele of *OsHKT1;5* that might be identical to some but not to all accessions of the recognized salt tolerant cultivars Pokkali and Nona Bokra.

## Conclusion

In this present study, seedling stage salt tolerance was evaluated on 204 rice accessions from the Bengal and Assam Aus Panel (BAAP) under control and salt stress conditions in hydroponic and soil systems. There were significant differences in genotypes, treatment effects and genotype × treatment interactions in most of these traits. With the exception of potassium content, corresponding traits were significantly correlated between hydroponic and soil systems under salt stress. Eight cultivars including the known tolerance genotype Pokkali were identified as highly salt tolerant. GWA mapping revealed a total of 97 and 74 QTLs in hydroponic and soil systems, respectively. A total of 94 of all QTLs identified here overlap with the previously salt QTLs giving good confidence that they truly affect these traits. Despite there being several recent GWA mapping studies on rice, 40 of the QTLs reported here have not been reported before. A total of 65 candidate genes were found in 52 QTLs, including a well-known major gene *OsHKT1;5* and two post-translational modifications_SUMOylation genes (*OsSUMO1* and *OsSUMO2*). The salt tolerance *aus* rice accessions identified in this study will be important resources for rice breeding program, while the QTLs and candidate genes will provide useful information for future studies in genetics of salinity tolerance in rice.

## Data Availability Statement

The raw data supporting the conclusions of this article will be made available by the authors, without undue reservation, to any qualified researcher.

## Author Contributions

AP and GN designed the experiment. CC conducted the experiments and performed data analysis. All authors wrote and approved the manuscript.

## Conflict of Interest

The authors declare that the research was conducted in the absence of any commercial or financial relationships that could be construed as a potential conflict of interest.

## Footnotes

1 http://rice.plantbiology.msu.edu/

## Supplementary Material

Supplementary Figure 1 Plants in the hydroponic system.

Supplementary Figure 2 Plants in the soil system. **(A)** The first week; **(B)** the second week.

Supplementary Figure 3 The correlation between mean of replicates and pooled samples in Na, K content and Na/K ratio.

Supplementary Figure 4 Frequency distribution of Na, K content, Na/K ratio and Salt injury score in BAAP population in hydroponic and soil systems. **(A)** Pokkali; **(B)** POKKALI; **(C)** IR 36; **(D)** IR 29.

Supplementary Figure 5 PCA for salt related traits in hydroponic and soil systems.

Supplementary Figure 6 Range of phenotypic variation in hydroponic system for different fastSTRUCTURE groups. Bar is 95% confidence interval.

Supplementary Figure 7 Range of phenotypic variation in soil system for different fastSTRUCTURE groups. Bar is 95% confidence interval.

Supplementary Figure 8 Genome-wide association for relative shoot biomass, relative root biomass, and relative total biomass in hydroponic system.

Supplementary Figure 9 Genome-wide association for relative total biomass in soil system.

Supplementary Figure 10 Na content in shoots for haplotypes in candidate genes *OsSUMO1* and *OsSUMO2*.

Supplementary Table 1 The cultivars and the mean of salt indices traits in this study.

Supplementary Table 2 Descriptive statistics of traits for the genotypes grown in hydroponic and soil systems.

Supplementary Table 3 The percentage contribution of genotype for salt indices traits to the observed variation in the hydroponic and soil systems.

Supplementary Table 4 One-way ANOVA for genetic effects in Na and K contents in hydroponic and soil systems.

Supplementary Table 5 Pearson correlation among different phenotypic traits in rice grown in hydroponic system.

Supplementary Table 6 Pearson correlation between phenotypic traits in rice grown in soil system.

Supplementary Table 7 Significant QTLs detected for salt related traits in rice grown in hydroponic system.

Supplementary Table 8 Significant QTLs detected for salt related traits in rice grown in soil system.

Supplementary Table 9 QTLs identified in this study were co-localized with genes reported to be involved in salt stress in rice.

Supplementary Table 10 Different studies of GWAS of salt tolerance in rice.

Supplementary Table 11 Comparison the positions of QTLs detected in this study with the previously reported significant SNPs/QTLs associated for salt tolerance using GWA mapping.

Supplementary Table 12 The haplotype status of *OsSUMO1, OsSUMO2*, and *OsHKT1;5*, from RiceVarMap in different cultivars.
