# Supplementary material for: Genome-Wide Association Mapping for Salt Tolerance of Rice Seedlings Grown in Hydroponic and Soil Systems Using the Bengal and Assam Aus Panel
Source: Front Plant Sci. 2020 Oct 23;11:576479. doi: 10.3389/fpls.2020.576479 (PMC7644878; doi:10.3389/fpls.2020.576479)
Supplement: Supplementary Figure 1 — Plants in the hydroponic system. [file Data_Sheet_1.PDF]

Supplementary Figures S1-10

Genome-Wide Association Mapping for Salt Tolerance of Rice Seedlings Grown in Hydroponic and Soil Systems Using the Bengal and Assam Aus Panel

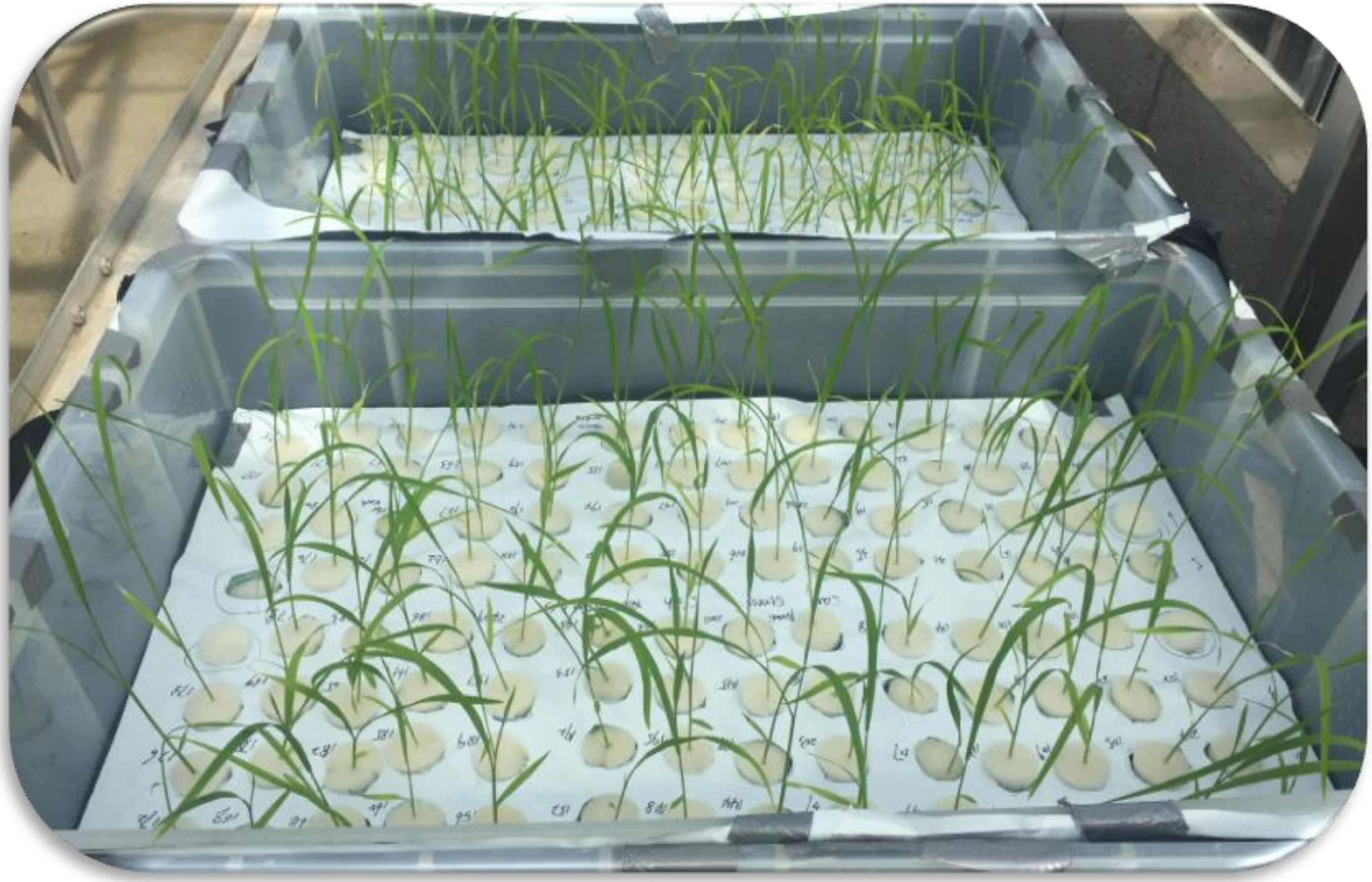

Supplementary Figure S1. Plants in the hydroponic system.

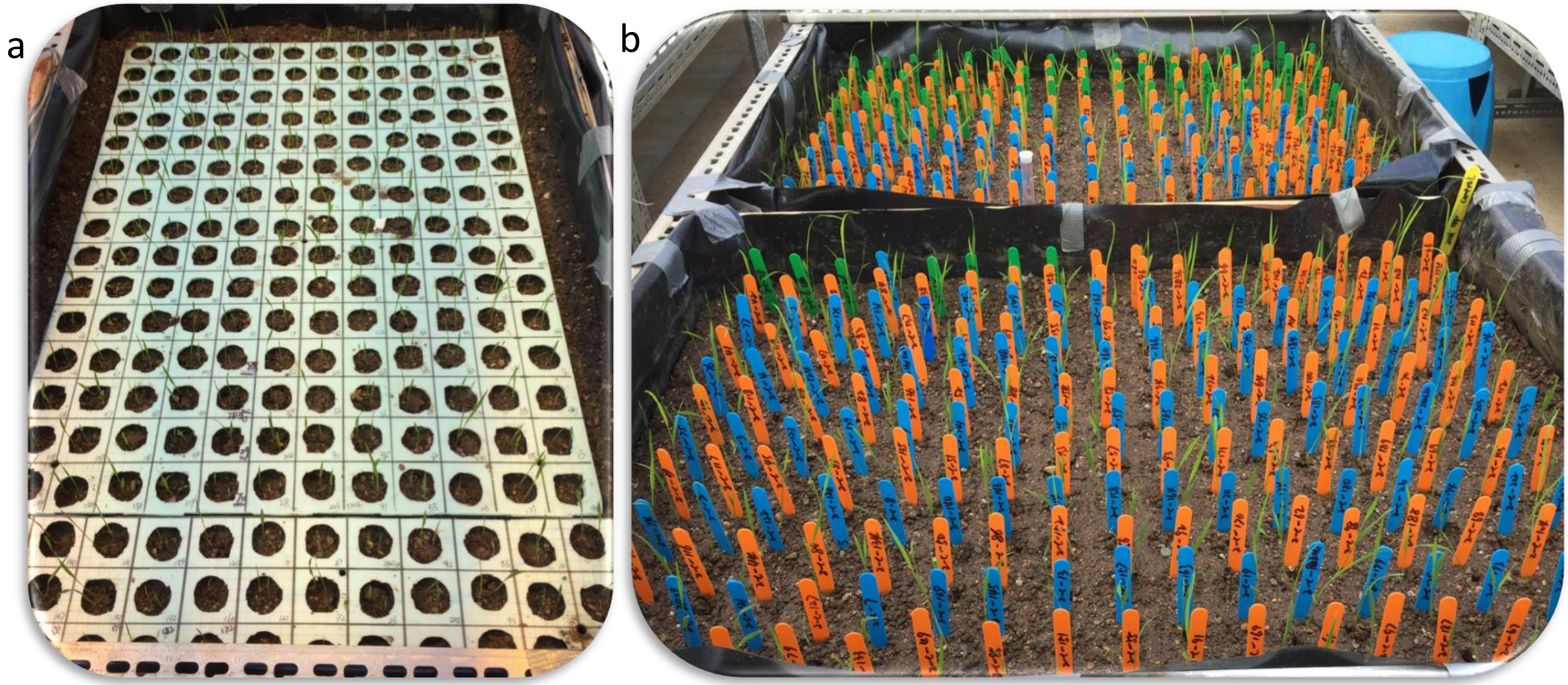

Supplementary Figure S2. Plants in the soil system. a, the first week; b, the second week.

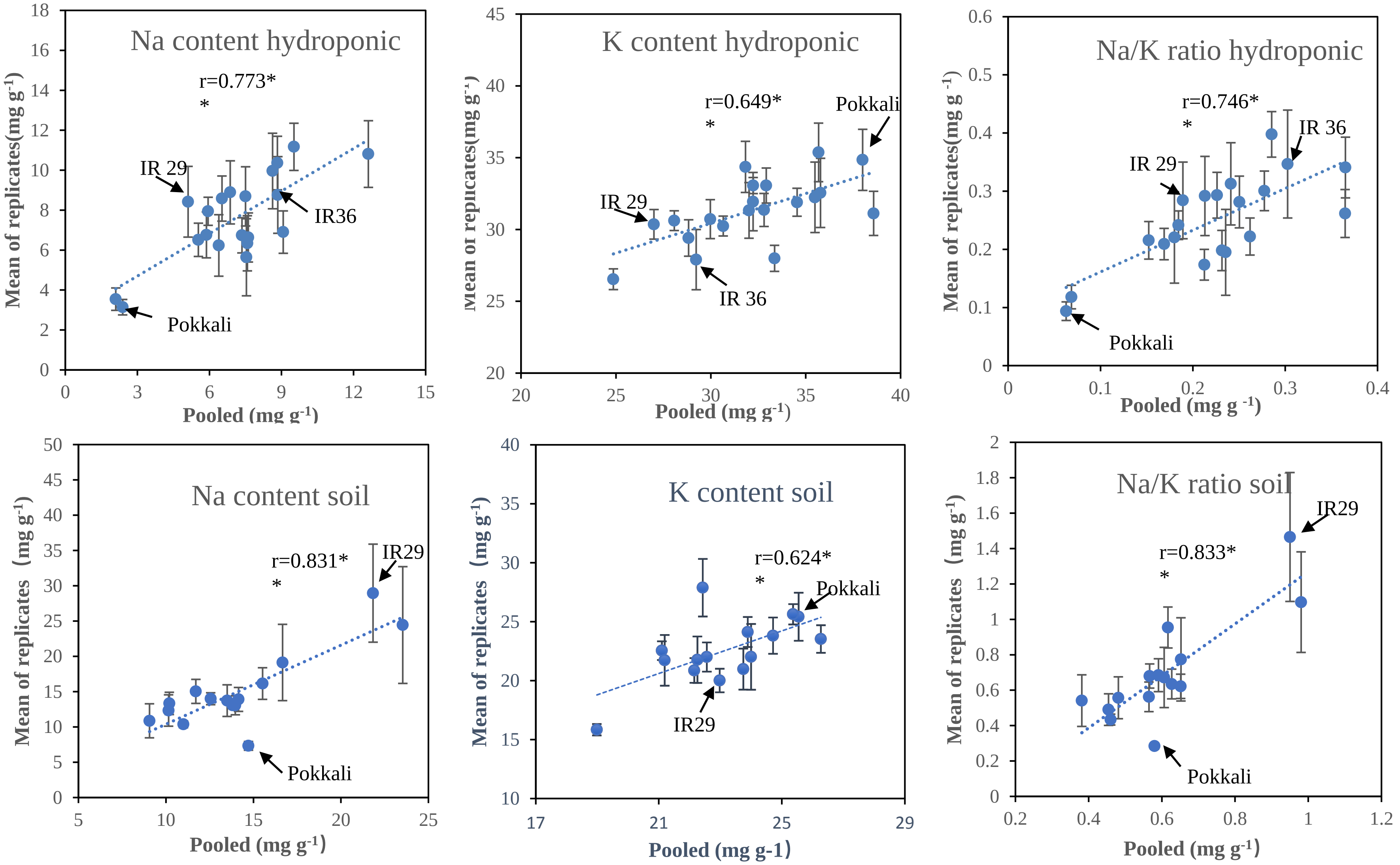

Supplementary Figure S3. The correlation between mean of replicates and pooled samples in Na, K content and Na/K ratio.

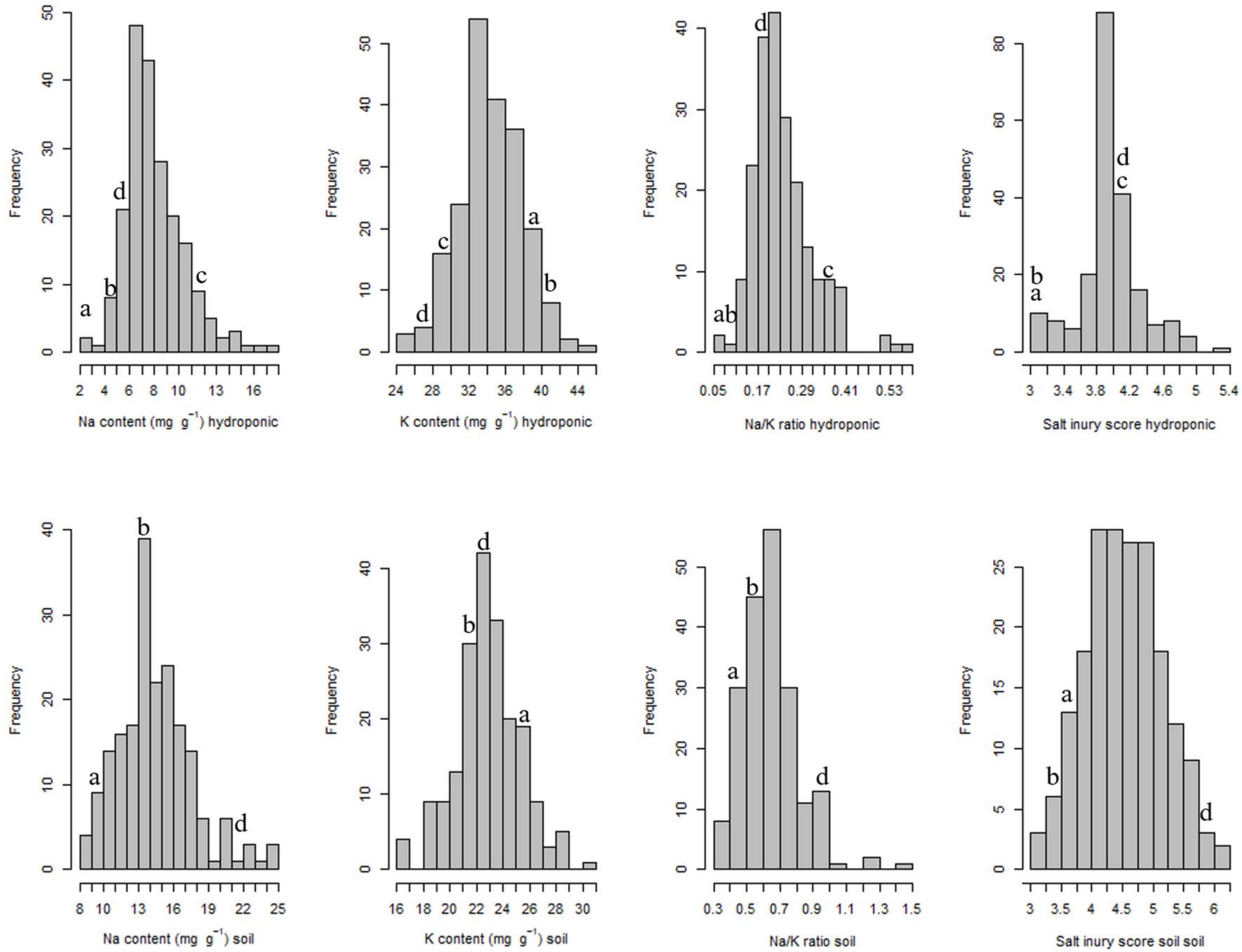

Supplementary Figure S4. Frequency distribution of Na, K content, Na/K ratio and Salt injury score in BAAP population in hydroponic and soil systems. a, Pokkali; b, POKKALI; c, IR 36; d, IR 29.

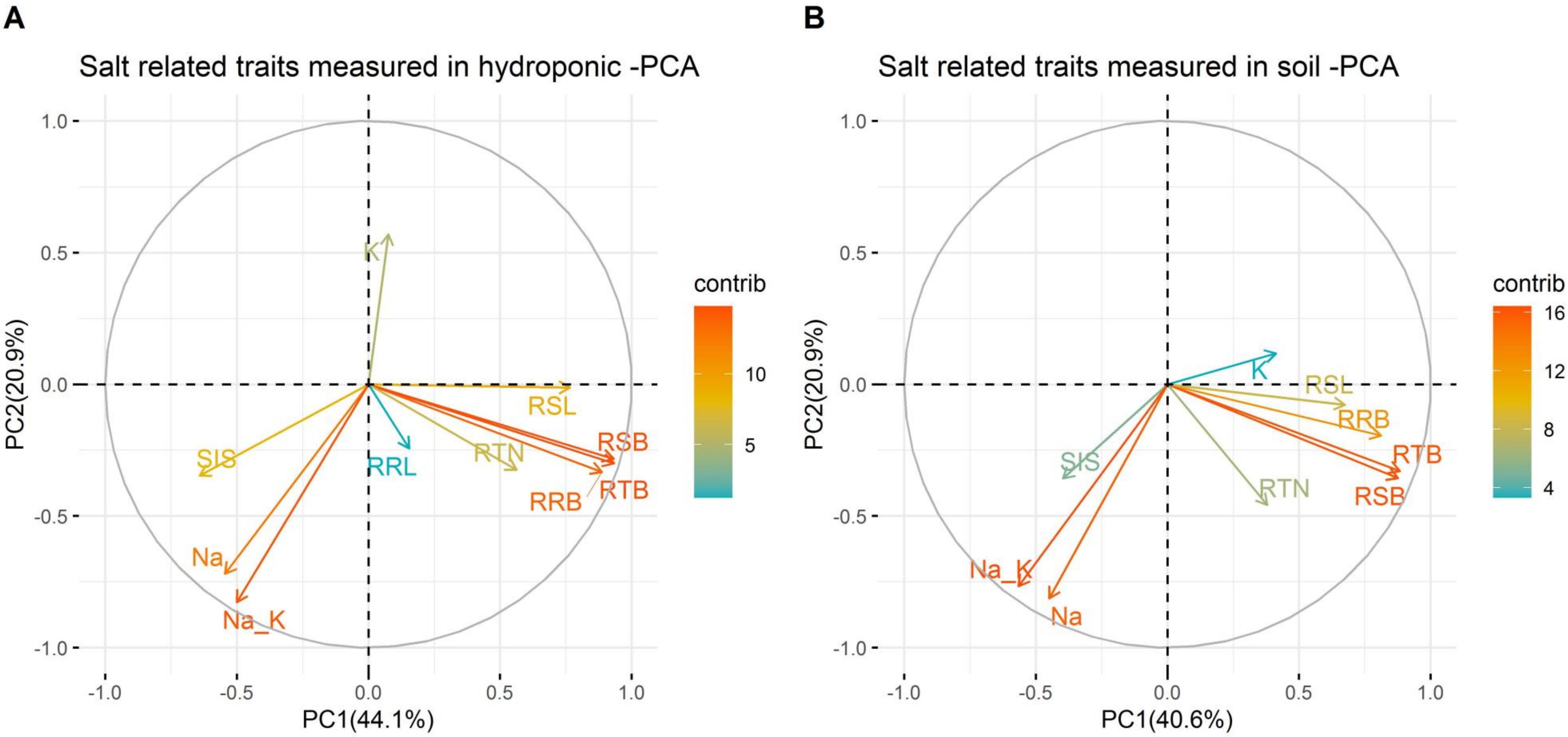

Supplementary Figure S5. PCA for salt related traits in hydroponic and soil systems. RSL: relative shoot length; RRL: relative root length; RSB: relative shoot biomass; RRB: relative root biomass; RSB:relative shoot biomass; RTB: relative total biomass; RTN: relative tiller number; SIS: salt injury score; Na_K: Na/K ratio

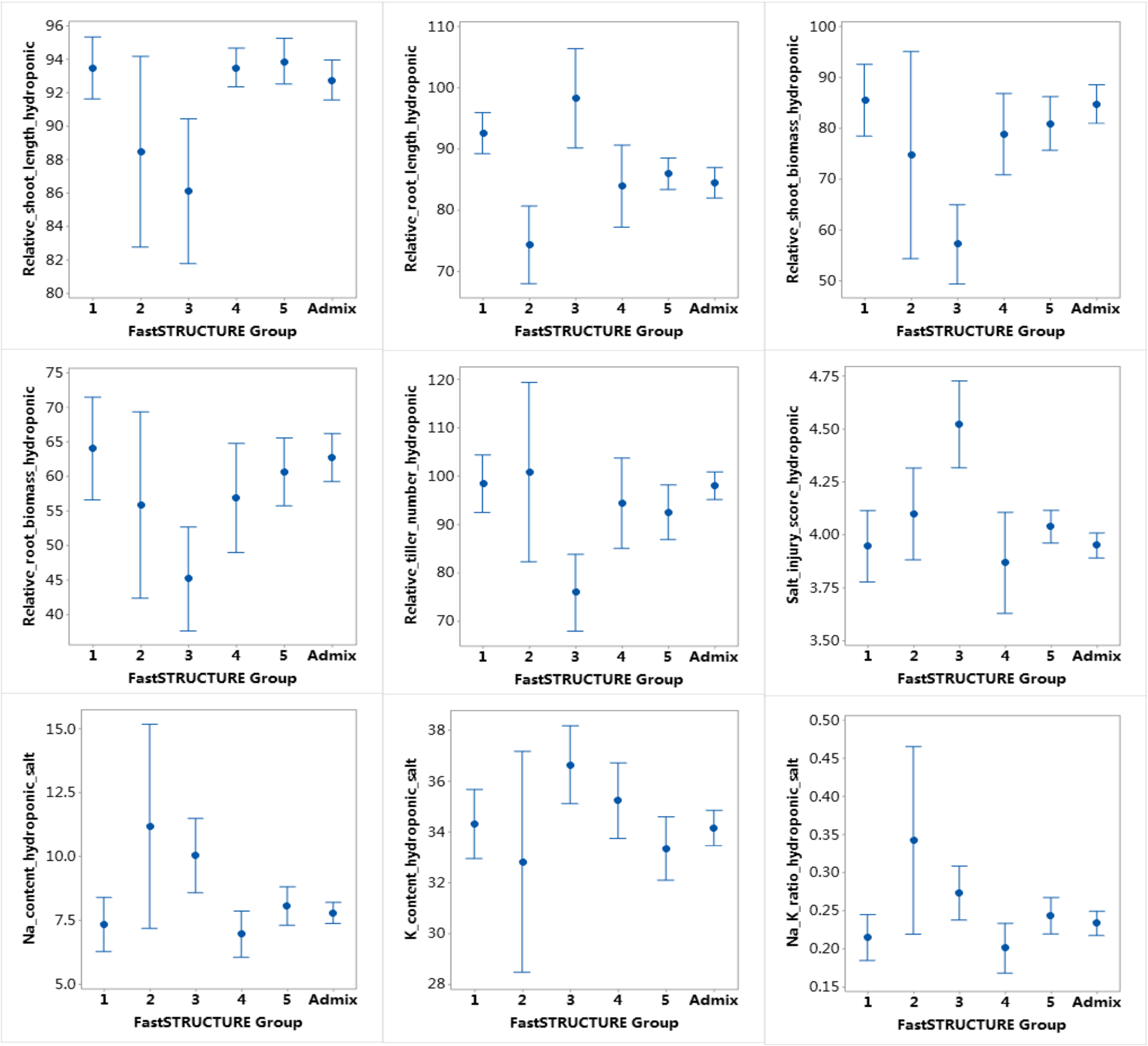

Supplementary Figure S6. Range of phenotypic variation in hydroponic system for different fastSTRUCTURE groups. Bar is 95% confidence interval.

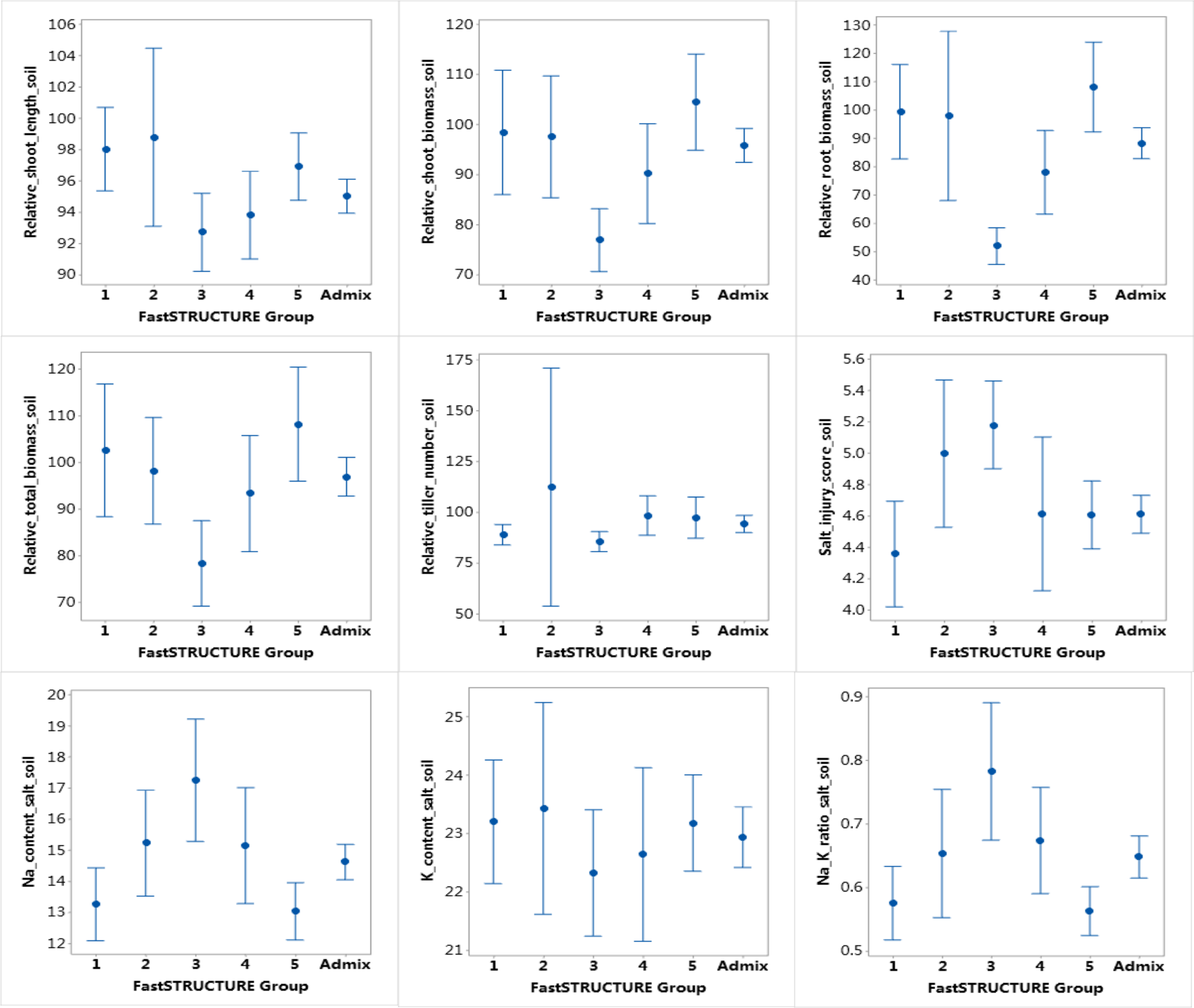

Supplementary Figure S7. Range of phenotypic variation in soil system for different fastSTRUCTURE groups. Bar is 95% confidence interval.

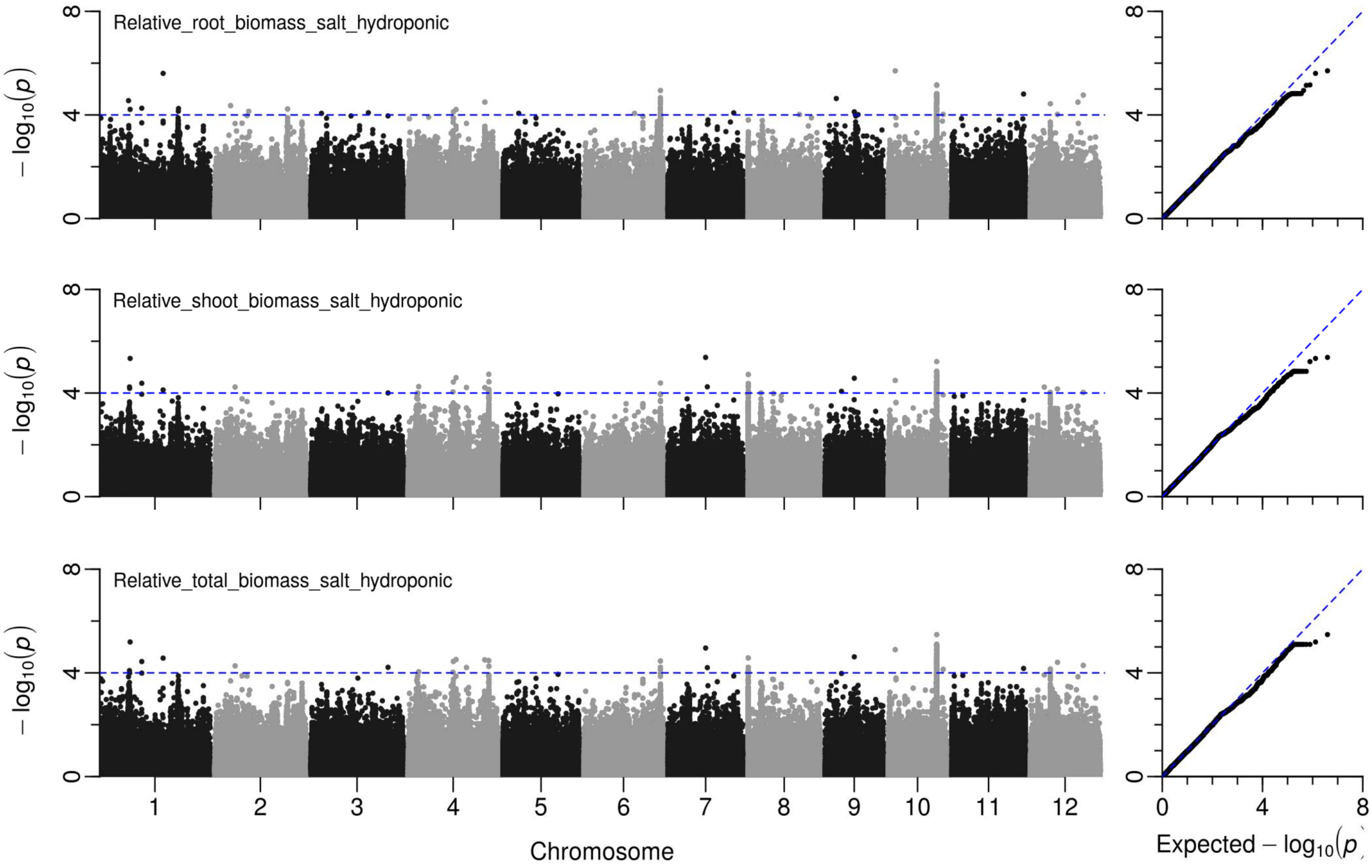

Supplementary Figure S8. Genome-wide association for relative shoot biomass, relative root biomass, and relative total biomass in hydroponic system.

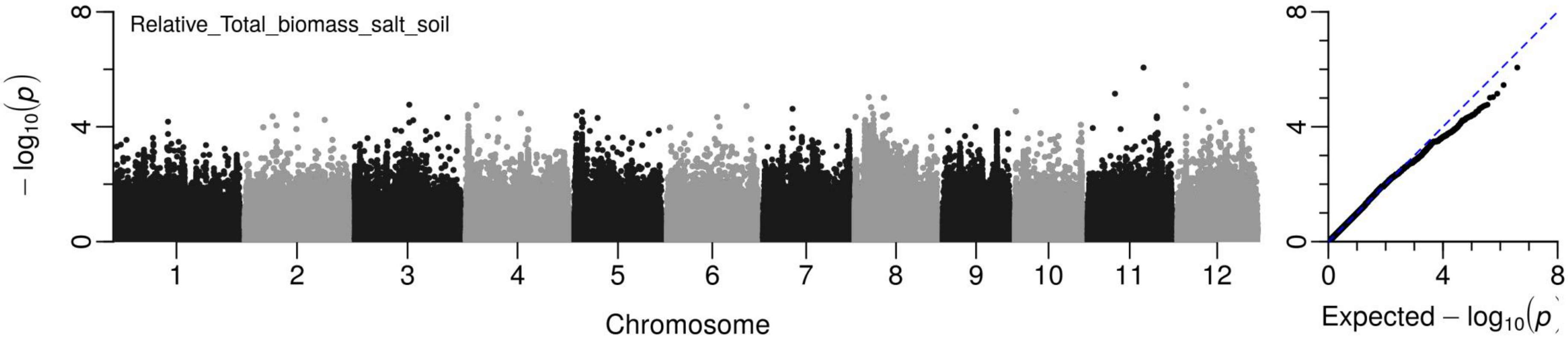

Supplementary Figure S9. Genome-wide association for relative total biomass in soil system.

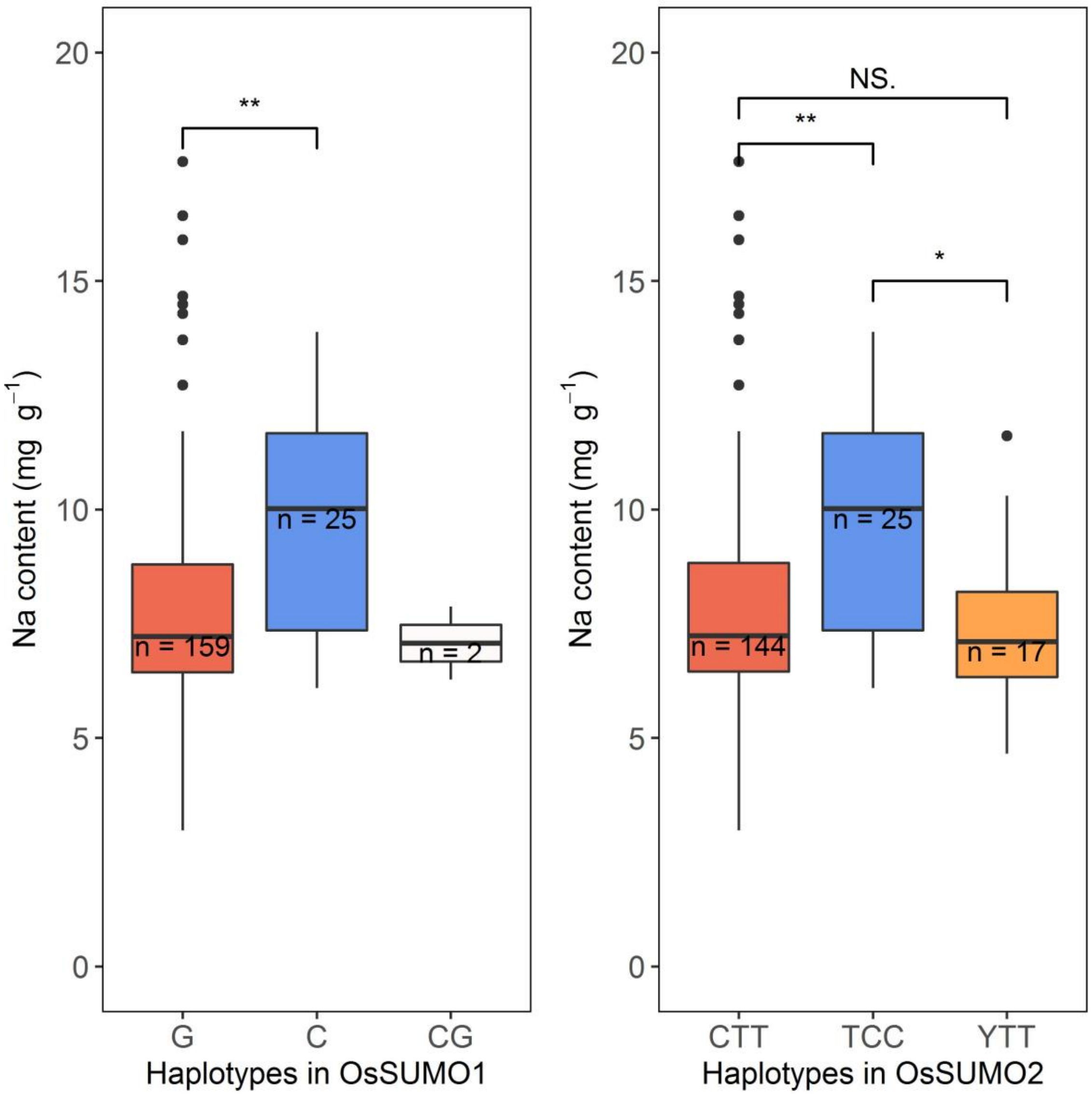

Supplementary Figure S10. Na content in shoots in hydroponic system for different haplotypes in candidate genes *OsSUMO1* and *OsSUMO2*.
